# Novel anti-tumor strategies: targeting the crosstalk between cancer stem cells and cancer-associated fibroblasts to resist drug resistance

**DOI:** 10.20517/cdr.2025.189

**Published:** 2026-01-20

**Authors:** Yang Shen, Yuting Sun, Xurui Li, Yi Wang, Ting Huang, Ting Li, Yi-Zhun Zhu, Lanlin Hu, Chuan Xu

**Affiliations:** ^1^School of Pharmacy, Faculty of Medicine, Macau University of Science and Technology, Macau 999078, China.; ^2^Yu-Yue Pathology Scientific Research Center, Chongqing 400039, China.; ^3^Jinfeng Laboratory, Chongqing 401329, China.; ^4^Department of Oncology & Cancer Institute, Sichuan Academy of Medical Science and Sichuan Provincial People’s Hospital, University of Electronic Science and Technology of China, Chengdu 610042, Sichuan, China.; ^5^Pathology Department, Shiyan Taihe Hospital, Shiyan 442000, Hubei, China.; ^6^Cancer Center, Medical Research Institute, Southwest University, Chongqing 400716, China.; ^7^Department of Radiation Oncology, Sichuan Clinical Research Center for Cancer, Sichuan Cancer Hospital & Institute, Sichuan Cancer Center, Affiliated Cancer Hospital of University of Electronic Science and Technology of China, Chengdu 610040, Sichuan, China.; ^#^These authors contributed equally to this work.

**Keywords:** Cancer stem cell, cancer-associated fibroblast, tumor microenvironment, stromal reprogramming, therapeutic resistance

## Abstract

The reciprocal feedback between cancer stem cells (CSCs) and cancer-associated fibroblasts (CAFs) is increasingly recognized as a driver of therapeutic resistance and tumor evolution. According to the “soil and seed” hypothesis, CAFs create a biochemical and biomechanical “soil” for CSCs to seed, grow, and thrive. In turn, CSCs manipulate and transform fibroblasts to promote CSC traits, thus completing the loop of CAF-CSC crosstalk through bidirectional molecular communication within the tumor microenvironment. This review encompasses recent advances in CAF heterogeneity, including conserved and malignancy-specific subtypes, as well as the molecular dialogue driving resistance. We also briefly discuss emerging therapeutic approaches, particularly the potential of natural compounds to target both CSCs and CAFs. By bridging mechanistic insights with translational innovations, this review provides a roadmap for breaking the CSC-CAF alliance, offering hope for overcoming therapeutic resistance and improving cancer outcomes.

## INTRODUCTION

The “soil and seed” theory, first articulated by Stephen Paget in 1889, suggests that metastatic cancer cell dissemination (“seeds”) relies on a permissive microenvironment (“soil”) to thrive. This concept has evolved to underscore the dynamic crosstalk between cancer stem cells (CSCs) and the tumor microenvironment (TME) in driving tumor progression and drug resistance.

A crucial advance in CSC biology occurred in 1997, when Bonnet and Dick identified leukemia stem cells (LSCs) in acute myeloid leukemia (AML), determining their capacity to recapitulate tumor heterogeneity upon transplantation^[1]^. This work laid the foundation for the formal CSC hypothesis in 2001. Subsequent breakthroughs, such as isolating CD44^+^ CD24^-^ tumor-initiating cells in breast cancer by Al-Hajj *et al.*, solidified the role of CSCs in solid tumors^[2]^. With the advancement of single-cell sequencing technology, CSC-specific transcriptional programs and niche interactions have been resolved, further validating their role as “seeds” of tumor progression and therapeutic resistance. CSCs exhibit plasticity, balancing quiescence with activation under microenvironmental cues to fuel tumor growth and drug resistance^[3,4]^.

The TME, or the “Soil”, is a dynamic ecosystem, shaped by tumor and stromal cells^[5]^. Fibroblasts, which are traditionally recognized for their role in wound healing and matrix synthesis^[6]^, constitute the predominant cellular component within the tumor stroma and are reprogrammed into cancer-associated fibroblasts (CAFs) within the TME. Activated CAFs secrete growth factors, cytokines, extracellular vesicles (EVs) containing various biological signals, and metabolites to support CSC self-renewal and treatment resistance^[7,8]^. Conversely, CSCs reciprocally activate CAFs via exosome microRNAs (miRNAs) and pro-fibrotic signals such as transforming growth factor-β (TGF-β) and interferon regulatory factor 6 (IRF6), driving extracellular matrix (ECM) remodeling and metabolic reprogramming to acidify the TME further and promote therapeutic resistance^[9-12]^.

Emerging evidence highlights a feedforward loop between CSCs and CAFs that drives tumor progression and therapeutic resistance. However, the molecular network governing this crosstalk remains poorly defined. We first provide a summary of CAF heterogeneity in various cancers, review recent advancements in the molecular mechanisms of CSC-CAF crosstalk, and discuss therapeutic strategies targeting this axis to disrupt the “vicious cycle” and overcome drug resistance.

## HETEROGENEITY OF CAFS

The spatial and temporal heterogeneity of CAFs underpins their multifaceted roles in remodeling the TME. Spatially, CAFs exhibit niche-specific functions such as ECM remodeling and angiogenesis. At the same time, their temporal plasticity, driven by reversible dedifferentiation in response to tumor-derived signals, enables adaptive shifts between activation states. This functional versatility, arising from diverse tissue origins and molecular drivers, further amplifies CAF heterogeneity and context-dependent contributions to tumor progression. Although single-cell technology facilitates the analysis of subtypes, accurately defining their roles remains a formidable challenge due to the absence of definitive molecular markers.

### Cellular origins of CAFs

CAFs arise from multiple precursors through tumor-driven reprogramming; their sources can be grouped into three main categories.

#### Activation of resident fibroblasts

The primary source of CAFs is the resident fibroblasts that are reprogrammed by TGF-β signaling, janus kinase (JAK)/signal transducer and activator of transcription (STAT), wingless/int (Wnt)/β-catenin signaling, cytokines, and miRNAs, including miR-31, miR-24, miR-155, and others^[13-15]^. Single-cell RNA (scRNA) reveals rich repeat containing protein 15 (LRRC15)^+^ myofibroblasts derive from peptidase inhibitor 16 (PI16)^+^/collagen type XV alpha 1 chain (COL15A1)^+^ progenitor-like fibroblasts and regulator of G-protein signaling 5 (RGS5)^+^ pericytes^[16]^.

#### Derivation from stem/progenitor cells

Beyond the activation of resident fibroblasts, various stem and progenitor cells can give rise to CAFs: Hypoxia triggers the expression of long non-coding RNA HIF1A antisense RNA 3 (lncRNA HIF1A-AS3), which facilitates the differentiation of mesenchymal stem cells into CAFs via miR-142-3p/miR-24-3p/PROX1/β-catenin signaling pathway and contributes to oxaliplatin resistance in gastric cancer^[17]^. A seminal study demonstrated that mouse-induced pluripotent stem cells (iPSCs) acquired CSC-like properties when exposed to conditioned medium (CM) from breast cancer cells. Remarkably, these CSC-like cells differentiated into alpha-smooth muscle actin (α-SMA)^+^; fibroblast-specific protein-1 (FSP1)^+^; Vimentin^+^ CAFs expressing pro-fibrotic mediators such as chemokine (C-X-C motif) ligand 12 (CXCL12), collagen type XI alpha 1 chain (COL11A1), and transforming growth factor beta 1 (TGF-β1), providing direct evidence that CSCs can directly generate CAFs within the TME^[18]^. Additionally, hematopoietic stem cell-derived fibroblast growth factor receptor 2 (FGFR2)^+^ fibrocytes differentiate into CAFs via the CXCL12/C-X-C chemokine receptor Type 4 (CXCR4) axis in esophageal cancer^[19]^.

#### Transdifferentiation from other mature cell types

Furthermore, other cell types of non-fibroblast lineages can transdifferentiate into CAFs, such as macrophages, stellate cells, and CSCs. In non-small cell lung cancer (NSCLC), macrophages transdifferentiate into CAFs via TGF-β1-mothers against decapentaplegic homolog 3 - runt-related transcription factor 1 (Smad-Runx1) signaling^[20]^, while hepatic stellate cells transition into CAFs via TGF-β1^[21]^. Hypoxia-induced calbindin 2 (CALB2) drives pancreatic stellate cell conversion into inflammatory CAFs (iCAFs)^[22]^. Moreover, lineage-tracing studies based on genetic pedigree tracing and fluorescent studies confirm the contributions from adipocytes, endothelial cells, mesenchymal stem/stromal cells, and epithelial cells. Challenges remain in defining CAF-specific markers and subtypes due to their heterogeneous cellular origins.

### Biomarkers and classification challenges

Defining fibroblasts has long been challenging due to their shared mesenchymal origin with adipocytes and other cell types, such as osteoblasts and chondrocytes^[23]^. Accordingly, cells identified as fibroblasts were typically characterized by their morphological features, such as an elongated, spindle-like phenotype with tapered ends and extended processes, along with their tissue location and the absence of markers specific to epithelial, endothelial, inflammatory, and leukocyte lineages. Recent advances in scRNA sequencing (scRNA-seq) and imaging techniques have revealed numerous proteins highly expressed in fibroblasts within the TME, which may serve as positive markers for identifying CAFs. These positive markers include α-SMA, FSP1, fibroblast activation protein (FAP), Vimentin, platelet-derived growth factor receptor α/β (PDGFRα/β), Podoplanin (PDPN), decorin (DCN), Tenascin-C (TN-C), Periostin (POSTN), Galectin-1 (Gal-1), Caveolin 1 (CAV1), and Ephs/ephrins^[24]^. However, these markers are not universally expressed across all CAF populations in diverse cancer types. The heterogeneous cellular origins and phenotypic plasticity of CAFs, combined with the limited specificity of currently available molecular markers for CAF identification, have collectively contributed to the emergence of multiple subtype classifications. These classifications are highly context-dependent, as nomenclature varies significantly across different tumor types and disease stages. Additionally, research on similar tumor subtypes remains in the early stages of development and lacks unified definitions. Initial investigations in pancreatic ductal adenocarcinoma (PDAC) have identified three distinct CAF subtypes: myofibroblastic CAFs (myCAFs), iCAFs, and antigen-presenting CAFs (apCAFs)^[25,26]^. Based on the single-cell omics, spatial location, gene expression patterns, and predicted functional roles, Cords *et al.* have revealed nine conserved CAF subtypes across breast, lung, colon, head and neck, and pancreatic cancer, reflecting their functional and spatial diversity within the TME. They are termed as matrix CAFs (mCAFs), iCAFs, vascular CAFs (vCAFs), tumor-like (tCAFs), interferon-response CAFs (ifnCAFs), heat shock protein high tCAFs (hsp_tCAFs), apCAFs, reticular-like CAFs (rCAFs), and dividing CAFs (dCAFs). Unfortunately, the lineage details concerning these various CAF types are presently insufficient^[27]^. Below, we present the main subtypes of CAFs, along with their functional attributes.

#### myCAFs and mCAFs

myCAFs were first identified in pancreatic cancer by Öhlund *et al.* in 2017, induced by TGF-β/mothers against decapentaplegic homolog (SMAD) signaling pathway and characterized by prominent α-SMA [actin alpha 2, smooth muscle (*ACTA2*)] expression and low interleukin (IL)-6 expression^[26]^. They formed distinct structural rings that surrounded and were adjacent to clusters of tumor cells, which drive tumor cell invasion and migration. One distinctive feature of myCAFs is their prominent synthesis of contractile proteins, including transgelin (TAGLN), matrix metallopeptidase (MMP) 11 (MMP11), myosin light chain 9 (MYL9), homeodomain-only protein homeobox (HOPX), POSTN, tropomyosin (TPM1/2), and myosin heavy chain 11 (MYH11)^[25,28]^. This unique trait forms a physical barrier that promotes tumor growth and contributes to treatment resistance. However, in a transgenic murine PDAC model, depletion of α-SMA^+^ myCAFs led to enhanced invasive capacity, activation of epithelial-mesenchymal transition (EMT), acquisition of stem-like characteristics, decreased overall survival (OS), and increased infiltration of CD4^+^Foxp3^+^ (Foxp3: forkhead box P3) regulatory T cells^[29]^. The absence of α-SMA^+^ myCAFs in the PDAC model resulted in tumors being unresponsive to gemcitabine therapy^[30]^. These studies demonstrate the pro- and anti-tumor effects of these CAFs. Additionally, they highly express adhesion-related genes, such as leucine rich repeat containing 15/17 (*LRRC15*/*17*) and asporin (*ASPN*), alongside EMT-associated genes [collagen triple helix repeat containing 1 (*CTHRC1*), *FAP*, inhibin beta A subunit (*INHBA*), *LRRC15*, matrix gla protein (*MGP*), *POSTN*, and versican (*VCAN*)], and migration-related genes [*POSTN*, sulfatase 1 (*SULF1*), *INHBA*, and *VCAN*]. Their unique cartilage-associated markers, MGP and BGN, distinguish them from other subtypes despite transcriptional overlaps. scRNA-seq further resolved myCAF heterogeneity into distinct functional subtypes in breast cancer^[31,32]^. These include extra-cellular matrix (ECM)-myCAFs, characterized by high expression of ECM-related genes; finally, pan-cancer scRNA-seq referred to the subpopulation as mCAFs, which projected in proximity to myCAFs, suggesting similar transcriptomic profiles^[28]^. TGF-β-myCAFs, enriched in TGF-β signaling; wound-myCAFs, associated with wound-healing pathways; acto-myCAFs, linked to actomyosin signaling; interferon α/β (IFNαβ)-myCAFs, reflecting IFNαβ responses. Notably, ECM-myCAFs and TGF-β-myCAFs dominate Luminal A tumors, correlating with an immunosuppressive TME enriched in cytotoxic T-lymphocyte associated protein 4 (CTLA4)^+^/programmed cell death protein 1 (PD-1)/T cell immunoglobulin and ITIM domains (TIGIT)^+^/CD4^+^ T cells and reduced CD8^+^ T cell infiltration, which contribute to primary immunotherapy resistance. In contrast, wound-myCAFs are associated with T-lymphocyte-rich niches and immuno-protective TME^[31,32]^. A new subtype of CAFs with a highly activated metabolic state (meCAFs) exhibits high expression of phospholipase A2 group IIA (PLA2G2A), cellular retinoic acid binding protein 2 (CRABP2), lactate dehydrogenase B (LDHB), and phosphoglycerate kinase 1 (PGK1), which promote tumorigenesis via translation, mitochondrial translation, glycolysis, noncanonical nuclear factor kappa-B (NF-κB) signaling, cyclin dependent kinase Inhibitor 1A/1B (P21/P27) degradation, and the myelocytomatosis oncogene (MYC) pathway in loose-type PDAC; these CAFs potentiate higher metastasis with poor clinical outcomes but better immunotherapy responses. Comparably, myCAFs with high expression of collagen type X alpha 1 chain (COL10A1), POSTN, and MMP11 promote tumor progression through integrin alpha V beta 3 (AVB3) integrin, focal adhesion, ECM-receptor interaction, protein tyrosine kinase 2 (PTK2), focal adhesion kinase (FAK), platelet-derived growth factor (PDGF), and mesenchymal-epithelial transition factor (MET) signaling pathways in dense-type PDAC^[33]^.

Pan-cancer scRNA analysis reveals analogous transcriptomic signatures between myCAF and mCAF, particularly in the expression of collagens and ECM constituents^[34]^. Despite this similarity, mCAFs exhibit sufficiently distinct features to be classified as a separate subgroup, including low expression of the canonical myCAF marker α-SMA and elevated expression of ECM-related genes such as *COL10A1*, *CTHRC1*, and *POSTN*^[34]^. mCAF subtype mainly expresses genes associated with ECM organization and collagen formation. While the myCAF subtype exhibits high expression of genes associated with the angiopoietin and PDGF pathways, it contributes to angiogenesis, contractile function, and myogenesis^[34]^. Single-cell pseudotime analysis suggests a developmental relationship between these subtypes, with mCAFs occupying intermediate stages and myCAFs emerging in the terminal phase, indicating a dynamic shift in CAF subpopulations during tumor progression^[34]^. Additional studies support the functional and spatial stratification of these subtypes. For example, in NSCLC, mCAFs harbor high levels of ECM genes [e.g., collagen type I alpha 1 chain (*COL1A1*), collagen type III alpha 1 chain (*COL3A1*), collagen type IV alpha 1 chain (*COL6A1*), collagen type X alpha 1 chain (*COL10A1*)] and are associated with strong EMT and TGF-β signaling. In contrast, myCAFs are marked by myogenesis-related genes such as *ACTA2*, myocyte enhancer factor 2C (*MEF2C*), *MYH11*, and integrin subunit alpha (*ITGA7*), mirroring the contractile myCAFs found in pancreatic cancer^[35]^. Similarly, in human intrahepatic cholangiocarcinoma (ICC), mCAFs show high expression of ECM components, including collagen type V alpha 1 chain (COL5A1), collagen type V alpha 2 chain (COL5A2), collagen type IV alpha 3 chain (COL6A3), Fibronectin (FN1), lumican (LUM), DCN, and VCAN^[36]^. The functional distinction between these subtypes is further highlighted by their clinical impact. Mouse models of pancreatic cancer revealed LRRC15^+^ myCAFs suppress CD8^+^ T cell infiltration and promote tumor progression, a parallel to mCAFs in Luminal A breast cancer^[37]^. Moreover, the work by Cords demonstrated that a high density of mCAFs correlates with poor patient prognosis, as these CAFs form a peritumoral barrier that impedes immune cell infiltration^[38]^. Other studies have also reported immunomodulatory functions for mCAFs in NSCLC; Salmon *et al.* have shown that loose fibronectin and collagen regions promote active T-cell motility in NSCLC by real-time imaging, while T-cell migration is significantly impaired in areas of dense ECM^[39]^. Treatment with collagenase D counteracted the immune-excluding phenotype by remodeling the stromal ECM. Consistent with this, ablating FAP-expressing cells in a transgenic mouse model decreased ECM production and T-cell retention within Lewis lung carcinoma stroma, a process mediated by interferon-γ (IFNγ) and tumor necrosis factor-α^[40]^. Later studies refined this taxonomy. Collagen type XIII alpha 1 chain (COL13A1)^+^/collagen type XIV alpha 1 chain (COL14A1)^+^-matrix fibroblasts were identified in early-stage lung adenocarcinoma (LUAD), and ACTA2^+^/TAGLN^+^ myCAFs were identified in advanced metastatic tumors, suggesting a possible link between these subtypes of myCAFs in advanced tumors^[41]^. TAGLN^+^ mCAFs correlated with lymph node metastasis, driving IL-6 secretion via NF-κB activation^[42]^. Spatial transcriptomics revealed that COL11A1^+^ CAFs were characterized by a pro-tumor myCAF phenotype, including ECM genes [*FAP*, *POSTN*, *CTHRC1*, and gremlin-1 (*GREM1*)] in NSCLC patients who were resistant to immune checkpoint blockade therapies. COL11A1^+^ CAFs via discoidin domain receptor tyrosine kinase 1 (DDR1) and partner with secreted phosphoprotein 1 (SPP1)^+^ macrophages to block CD8^+^ T cells through SPP1-CD44^[43]^; POSTN^+^ myCAFs in close localization with SPP1^+^ macrophages and were associated with immunotherapy resistance in NSCLC^[44]^. POSTN^+^ mCAFs in HCC create exclusionary niches for T cells, such as ECM-myCAFs in breast cancer (BC)^[45]^. Wong *et al.* identified the CTHRC1^+^ ASPN^+^ FAP^+^ endoglin (ENG)^+^ CAF subtype in ICC and intraductal carcinoma that drives angiogenesis and suppresses cytotoxic T cell activity, mirroring the pro-angiogenic vCAFs and myCAFs^[46]^. In gastric cancer, through sub-clustering analysis, three distinct subpopulations of mCAFs were identified: POSTN^+^ mCAFs marked by LUM and POSTN, cystatin SN (CST1)^+^ mCAFs marked by LUM and CST1, secreted frizzled related protein 1 (SFRP1)^+^ mCAFs marked by LUM and SFRP1, myosin light chain kinase (MYLK)^+^ vCAFs characterized by mesothelial and mesenchymal cell marker (MACM) and MYLK, and RGS5^+^ vCAFs marked by MACM and RGS5. Li *et al.* identified two primary CAF subgroups via scRNA-seq. CAF A cluster (CAF-A) is enriched in ECM-remodeling genes (*MMP2*, *DCN*, and *COL1A2*), while CAF B cluster (CAF-B) is marked by myofibroblast markers (ACTA2, TAGLN, and PDGF)^[47]^. EMT-associated genes were broadly upregulated in CAFs, though their subtype specificity remains unresolved. Further studies identified thrombospondin 2 (THBS2)^+^ CAFs in colorectal cancer (CRC), which secrete collagen type VIII alpha 1 chain (COL8A1) to activate EMT and confer oxaliplatin resistance. In ovarian cancer, TGF-β-CAFs, marked by PSTN, ACTA2, and COL11A1, mirror the ECM-myCAFs and TGF-β-myCAFs of BC in their ECM remodeling functions, but lack immune-exclusion links, suggesting context-dependent functional divergence^[48]^. In high-grade serous ovarian cancer (HGSOC), mCAFs express Wnt family member 7B (WNT7B), transforming growth factor beta 3 (TGFB3), α-SMA, vimentin, COL3A1, COL10A1, and MMP11, directly promoting EMT via mechanisms shared with mCAFs in NSCLC^[49]^.

#### iCAFs

iCAFs, initially characterized by Öhlund *et al.* in pancreatic cancer, demonstrated low α-SMA expression, high IL-6 production, distal localization from tumor cells, and loss of myofibroblastic characteristics^[26]^. Later, their organoid and mouse models showed that pancreatic tumor-derived TGF-β/IL-1 dynamically regulate myCAFs-iCAFs interconversion^[50]^. These CAFs exhibit unique expression of a phospholipase *PLA2G2A*, complement pathway genes complement factor D (*CFD*) and complement component 3 (*C3*), and cytokines and chemokines *CXCL12*/*14*, alongside CD34, an inflammation and hematopoietic stem cell marker. Cords *et al.* identified CD34^+^ iCAFs adjacent to vascular structures, C-C motif chemokine ligand 21 (CCL21)^+^ CAFs encircling tertiary lymphoid structures, and ifnCAFs in proximity to tumor cells. The distinct spatial organization of CAFs exhibiting different inflammatory gene programs suggests that local microenvironmental cues and cellular interactions may determine phenotypic specialization among iCAF subgroups. Based on these observations, they propose that these three populations (CD34^+^, CCL21^+^, and ifnCAFs) represent distinct iCAF subtypes^[27,28]^. In the MMTV-PyMT (mouse mammary tumor virus-polyoma middle tumor-antigen) mammary tumor model, a subset of involution iCAFs displays elevated expression of involution-associated genes, specifically *Col1a1*, *Cxcl12*, and *Mmp3*^[51]^. Furthermore, IfnCAFs secrete interferon-response chemokines [*CXCL9*/*10*/*11*, indoleamine 2,3-dioxygenase 1 (*IDO1*)], as well as chronic inflammation markers such as IL-32. Gene set enrichment analysis (GSEA) demonstrates that iCAFs are characterized by upregulation of the IL6-JAK-STAT3 pathway, along with Kirsten rat sarcoma viral oncogene homolog (KRAS) signaling and complement signaling^[28]^.

Furthermore, IFNγ-iCAFs, detox-iCAFs, and IL-iCAFs were identified in breast cancer, implicating IFNγ signaling, detoxification, and interleukin-mediated pathways^[31,32]^. detox-iCAFs and IL-iCAFs are prevalent in triple-negative breast cancer (TNBC) and positively correlate with CD8^+^ T cell infiltration^[31,32]^.

Additionally, the formation of iCAFs is driven by multiple mechanisms, including activation of the JAK/STAT pathway by tumor-derived IL-1^[50]^, hypoxia in the TME^[52,53]^, and induction by IL-17A derived from interleukin-17-producing cytotoxic T cell (Tc17) cells^[54]^. In PDAC, higher levels of iCAFs promote chemoresistance by upregulating metallothioneins^[55]^, and the population of iCAFs increases in oxaliplatin-resistant PDAC patients. At the same time, the abundance of myCAFs remains unchanged^[56]^. Beyond their tumor-promoting functions in pancreatic cancer, iCAFs contribute to cancer progression in other malignancies: in breast cancer, they recruit bone marrow cells through CXCL12-dependent mechanisms and enhance MMP activity, thereby increasing tumor invasiveness^[57]^; in liver cancer, iCAFs drive tumor growth via the hepatocyte growth factor (HGF)-MET signaling pathway^[58]^; and in gastric cancer, they upregulate stemness-related pathways, including NF-κB signaling, tumor necrosis factor signaling, and cytokine-receptor interactions, underscoring their role in CSC biology^[59]^. Although iCAFs are predominantly associated with pro-tumorigenic functions across multiple studies, a distinct subpopulation of tumor-restrictive iCAFs, characterized by elevated osteoglycin expression, has been identified in pancreatic cancer^[60]^. In gastric cancer, iCAFs exhibited elevated levels of inflammatory cytokines and chemokines, such as IL-1β, IL-11, interleukin 13 receptor subunit alpha 1 (IL-13RA1), IL-24, MME (CD10), alanyl aminopeptidase, membrane (ANPEP, CD13), and CXCL1/3/8, as well as growth factors such as Wnt family member 2/5 (WNT2/5) and amphiregulin (AREG). Pathway analyses using Kyoto Encyclopedia of Genes and Genomes (KEGG) and pathway responsive genes for activity inference (PROGENy) disclosed that iCAFs were enriched in the IL-6 signaling pathway, hypoxia responses, and tumor growth signals, encompassing epidermal growth factor receptor (EGFR), WNT, mitogen-activated protein kinase (MAPK), and TGF-β, all vital for maintaining CSCs. Moreover, the vascular endothelial growth factor-vascular endothelial growth factor receptor (VEGF-VEGFR) signaling pathway, related to tumor angiogenesis, was implicated in both iCAFs and CST1^+^ mCAFs, suggesting their significance in advancing tumor invasion and metastasis. Trajectory examination of CAF clusters indicated that iCAFs emerged from CST1^+^ mCAFs via POSTN^+^ mCAFs, while another pathway showed RGS5^+^ vCAFs differentiating from CST1^+^ mCAFs through MYLK^+^ vCAFs. Transcriptomic similarity and differentiation trajectory assessments confirmed that CST1^+^ mCAFs acted as a transitional state governing cellular differentiation routes. Pseudo-temporal analysis of expression dynamics revealed increased activity of iCAFs’ signature genes, including *IL-11*, *IL-24*, *CXCL1*, *AREG*, twist family BHLH transcription factor 1 (*TWIST1*), and Wnt family member 5A (*WNT5A*), corroborating the differentiation trajectory results. Significantly, IL-11^+^ CD10^+^ iCAFs were recognized as crucial components of the CSC niche by spatial and single-cell transcriptomic, enhancing tumor stemness by upregulating SRY-box transcription factor 9 (SOX9) and olfactomedin 4 (OLFM4), which induce Lapatinib resistance through the AREG-erb-b2 receptor tyrosine kinase 2 (ERBB2) signaling mechanism^[61]^. In ovarian cancer, IL-1-CAFs, enriched in tumors with immune infiltration, secrete CXCL12/14 and C-C motif ligand 2 (CCL2), and express suppressor of cytokine signaling 3 (SOCS3), potentially recruiting CD8^+^ T cells via CXCR4, resembling IL-iCAFs and detox-iCAFs in breast cancer^[48]^.

#### apCAFs

The apCAFs that characterized MHC Class II molecules (MHC-II) molecules [HLA class II histocompatibility antigen, DR alpha chain (HLA-DRA), HLA class II histocompatibility antigen, DR beta chain 1 (HLA-DRB1) and invariant chain of MHC class II (CD74)] were initially unveiled by Elyada *et al.* through scRNA-seq analysis and multiplexed imaging techniques in PDAC^[25]^. Unique CAFs have been classified as apCAFs due to their exceptional capacity to stimulate CD4^+^ T cells in a manner specific to antigens. In pancreatic cancer, apCAFs may derive from mesothelial cell transformation induced by IL-1 and TGF-β. These CAFs can directly engage with naïve CD4^+^ T cells, promoting the formation of regulatory T cells, consequently contributing to tumor growth, immunosuppression, and immunotherapy resistance^[62]^. Conversely, in lung cancer, reducing apCAF levels through MHC-II removal paradoxically increases tumor burden, decreases survival rates, and diminishes T cell infiltration, suggesting an unexpectedly protective role for apCAFs against tumor progression in this context^[63]^. In gastric cancer, apss are strategically situated in proximity to tertiary lymphoid structures, bolstering T cell-mediated anti-tumor immunity by T cell activation. Notably, a greater infiltration of apCAFs is associated with enhanced responsiveness to immunotherapy; similarly, analogous outcomes were noted in TNBC patients undergoing immunotherapy^[64]^. Beyond antigen presentation and processing, apCAFs demonstrate upregulation of pathways involving fatty acid metabolism, MYC targets, and mammalian target of rapamycin complex 1 (MTORC1) signaling^[25]^. However, it is important to note that apCAFs are infrequently observed in most cancer types. In addition, precise localization within the TME remains challenging due to the difficulty in differentiating them from abundant MHC-II^+^ antigen-presenting cells in tissue. Although Elyada *et al.* employed morphological criteria to identify apCAFs and address this limitation, they did not identify the preferential location of these cells in the TME^[25]^. Recently, Zhang *et al.* revealed that apCAFs were in close spatial proximity to cancer cells in gastric cancer, and pericytes serve as a crucial source for the formation of apCAFs, mCAFs, and iCAFs^[65]^. The exact function of apCAFs remains controversial; consequently, further investigation is required to elucidate their spatial and functional relationships with other TME components and to assess their potential therapeutic relevance.

#### Additional CAF subtypes

Bartoschek *et al.* identified four subtypes of CAFs in the transgenic mouse model of breast cancer, including vCAFs, cCAFs, mCAFs, and dCAFs^[66]^. vCAFs display high levels of *NOTCH3*, *COL18A1*, and *MCAM* (encoding CD146); some of these cells express pericyte marker RGS5, but RGS5 is not one of the top differentially expressed genes in this cluster. MYLK^+^ vCAFs are characterized by MACM and MYLK, and RGS5^+^ vCAFs are marked by MACM and RGS5 in gastric cancer. Zhang *et al.* demonstrated that ICC cell-derived miR-9-5p upregulates IL-6 secretion in CD146^+^ vCAFs to promote tumor progression^[36]^. The exact role of those cells is still unclear; however, given their perivascular localization and expression characteristics, they may play a role in angiogenesis^[27,28]^.

tCAFs express hypoxia marker carbonic anhydrase IX (*CAIX*), suggesting their proximity to tumor-derived hypoxic regions. They also express genes associated with proliferation, migration, and metastasis, including *PDPN*, *MME* (encoding CD10), 5′-nucleotidase ecto (*NT5E*, encoding for CD73), transmembrane protein 158 (*TMEM158*), N-myc downstream regulated 1 (*NDRG1*), and factors such as enolase 1 (*ENO1*) and glyceraldehyde-3-phosphate dehydrogenase (*GAPDH*)^[28]^. In 2024, Cords *et al.* reported lung-specific tCAFs defined by upregulated glycolysis and CD10/CD73 expression and heat shock proteins correlated with poor prognosis^[38]^. CD10 expression in tumor stroma and CAFs has been linked to tumor stemness, chemoresistance in NSCLC, as well as high tumor grades and poor patient survival^[67]^. Due to their gene expression profile resembling that of tumor cells, which correlates with poor patient prognosis and appears to support tumor progression in NSCLC, they are termed tCAFs^[38]^. A subset of tCAFs in breast cancer, termed hsp_tCAFs, expresses heat-shock proteins *HSPH1* and heat shock protein 90 alpha family class A member 1 (*HSP90AA1*), showing metabolic adaptation to hypoxia. Gene set analysis shows enrichment of the glycolysis hallmark pathway in this CAF type. Similarly, the CAF subtype of cluster 5/7 diverged metabolically, exhibiting mammalian target of rapamycin (mTOR) activation and glycolytic reprogramming reminiscent of the hypoxic hsp-tCAFs in BC^[35]^. Alternative names for these cells include energy CAFs, metabolic CAFs, or glycolytic CAFs, but a more functionally descriptive term may be appropriate as further insights emerge.

rCAFs promote naïve T cell homing through the secretion of C-C motif chemokine ligand 19 (CCL19) and C-C motif chemokine ligand 21 (CCL21), mirroring the functions of lymphoid stromal cells.

dCAFs, previously termed cycling CAFs (cCAFs) and defined by cell cycle regulators tubulin alpha 1b (TUBA1B) and marker of proliferation Ki-67 (MKI67), represent a transient proliferative population^[66]^.

These subtypes drive tumor progression and therapeutic resistance with TME. However, classification inconsistencies persist due to phenotype overlap, observer dependence, and tumor-type-specific nomenclature. However, there are currently no clear factors that can distinctly define the pro-tumor or anti-tumor effects of CAFs. We will summarize heterogeneity in CAF subtypes across diverse malignancies, emphasizing their phenotypic and functional distinctions within distinct TMEs [Tables 1 and 2].

**Table 1:** Summary of the main CAF subtypes and features in various cancers

**Cancer**	**Model**	**Marker gene**	**Role in cancer**	**Ref.**
**myCAFs and mCAFs**				
Pancreatic cancer	Human	*ACTA2*, *POSTN*, *MMP11*, *COL1A1*, *FAP*	1. Promote immunotherapy resistance by impeding T cell infiltration 2. Promote invasion, metastasis, and proliferation by ECM remodeling, angiogenesis, focal adhesion, and contraction	[25,26,33]
	Kras^+/LSL-G12D^ Trp^53+/LSL-R17H^ KPC mouse model	*Acta2*, *Tgfb1*, *Col1a1*, *Fap*		[25,26,68]
	Mouse model of liver metastasis	*Acta2*, *Col1a1*, *Col3a1*		[69]
Breast cancer	Human	*FAP*, *POSTN*, *ANTXR*, *COL1A2*, *MMP11*		[28,31]
	Mouse allograft model of 4T1	*Col14a1*, *Vcan*, *Lum* *Fbn1*, *Smoc2*, *Loxl1*		[66]
Lung cancer	Human	*ACTA2*, *FAP*, *MMP11*, *COL3A1*, *COL1A1*, *POSTN*, *TAGLN*, *CTHRC1*		[35,38,41,70-72]
ICC	Human	*POSTN*, *COL6A3*, *FN1*		[36]
Gastric cancer	Human	*SFRP1 MMP1*, *POSTN*, *LUM*, *SFRP1CST1*		[61,64,65]
Colorectal cancer	Human	*ACTA2*, *MMP2*, *COL1A2*, *TAGLN*		[73]
	Human CRC liver metastasis	*COL1A1*, *COL3A1*, *COL6A3*		[69]
	Mouse model of liver metastasis	*Acta2*, *Col1a1*, *H2Q4*		[69]
Bladder urothelial carcinoma	Human	*MYH11*, *MYL9*, *RGS5*		[74]
Melanoma	Mouse	*Acta2*, *Postn*		[75]
Ovarian cancer	Human	*ACTA2*, *POSTN*, *MMP1*, *COL10A1*, *CTHRC1*		[48,49]
Soft-tissue sarcomas	Mouse	*Col12a1*, *Thbs2*, *Tnc*, *Cd74*, *Slpi*		[76]
**Inflammatory and immune-regulatory CAFs**				
Pancreatic cancer	Human	*IL-6*, *IL-8*, *CXCL12*, *PDGFRA*, *AGTR1*, *CXCL1*, *HAS1*	1. Promote the proliferation, survival, and metastasis of cancer cells through the process of angiogenesis 2. Promote proliferation through inflammation and chemotaxis 3. Promote the metastasis of cancer cells through metabolic and immunosuppression 4. Promote tumor stemness contributes to drug resistance	[25,26,77]
	Kras^+/LSL-G12D^ Trp^53+/LSL-R17H^ KPC mouse model	*Il-6*, *Cxcl12*, *Ly6c1*, *Cxcl1*, *Clec3b Col14A1*, *Igf1*, *Pdgfra*		[25,26]
	Mouse model of liver metastasis	*H2Q4*, *H2Q7*, *Hgf*, *Ifitm*		[69]
Breast cancer	Human	*IL6*, *CXCL12*, *CXCL14*, *DLK1*, *IGF1*		[31]
	PyMT/WT, PyMT/ELF5 mouse model	*Il6*, *Ly6c1*, *Cxcl1*, *Cxcl12*, *Ccl7*, *Clec3b*		[51,78]
Lung cancer	Human	*CXCL12*, *CXCL14*, *PDGFRA*, *TNXB*, *IL6*, *CXCL1*, *APOD*		[38,41,70,72,79]
ICC	Human	*FBLN1*, *IGF1*, *C3*, *SAA1*, *IL6*, *CXCL12*		[36,58]
	YAP/AKT, KRAS/p19 mouse models	*Cxcl12*, *Il6*, *Igf1*		[58]
Gastric cancer	Human	*IL11*, *IL24*, *MME*, *ANPEP*, *AREG*		[61,64,65]
Colorectal cancer	Human CRC liver metastases	*ISYNA1*, *NDUFA4L2*, *RGS5*		[69]
Bladder urothelial carcinoma	Human	*CXCL12*, *CXCL14*, *IL-6*, *PDGFRA*, *LOXL2*		[74]
Melanoma	Mouse	*Cxcl12*, *Pdgfa*, *Il6ra*		[75]
Ovarian cancer	Human	*CXCL12*, *CXCL14*		[48]
Soft-tissue sarcomas	Mouse	*Ly6c1*		[76]
**apCAFs**				
Pancreatic cancer	Human	*CD74*, *HLA-DRA*, *HLA-DPA1*, *HLA-DQA1*	1. Promote tumorigenesis in TME 2. Promote immune regulation and antigen presentation	[25]
	KPC mouse	*Cd74*, *H2-Ab1*, *H2-Aa*, *Saa3*		[25]
Breast cancer	Human	*HLA-DRA*, *HLA- DRB1*, *CD74*		[28,31,78]
	Mouse	*Cd74*, *Slpi*, *H2-Abl*, *H2-Aa*, *Eb1*		[31,78]
Lung cancer	Human	*CD74*, *HLA-DRB1*, *SLPI*, *HLA-DRA*		[70,79]
	Mouse	*Cd74*, *Slpi*		[80]
ICC	Human	*CD74*, *HLA-DRA*, *HLA-DRB1*		[36,58]
	Mouse	*H2-Q4*		[36,58]
Gastric cancer	Human	*CD74*, *HLA-DRB1*, *HLA-DRA*, *HLA-DPA1*	Augment T cell-mediated anti-tumor immune response and are associated with a favorable prognosis	[64,65]

KPC model-mouse model of pancreatic cancer (cell line-derived xenograft); PyMT/WT mouse model, mouse mammary tumor model; PyMT/ELF5 mouse model, a mammary restricted (MMTV) doxycycline-inducible (rtTA) Elf5 PyMT model; *Setd2*-deficient KSC model-mouse model of PDAC (*Setd2*-deficient). Ref.^[25]^ encompasses both human and animal models. CAF: Cancer-associated fibroblast; myCAFs: myofibroblastic CAFs; mCAFs: matrix CAFs; KPC: KrasG12D; Trp53R172H; Pdx1-Cre mouse model of pancreatic ductal adenocarcinoma; ICC: intrahepatic cholangiocarcinoma; CRC: colorectal cancer; ECM: extracellular matrix; PyMT/WT: polyomavirus middle T antigen/wild-type mouse model of breast cancer; PyMT/ELF5: polyomavirus middle T antigen/E74 like ETS transcription factor 5 mouse model of breast cancer; YAP: Yes1-associated transcriptional regulator; AKT: protein kinase B; KRAS: Kirsten rat sarcoma viral oncogene homolog; apCAFs: antigen-presenting CAFs; TME: tumor microenvironment; MMTV: mouse mammary tumor virus promoter-driven polyoma middle T antigen mouse model; KSC: KrasG12D; Smad4flox/flox; Cdkn2aflox/flox mouse model of pancreatic ductal adenocarcinoma; PDAC: pancreatic ductal adenocarcinoma; ACTA2/Acta2: actin alpha 2, smooth muscle; AREG: amphiregulin; Ccl7: C-C motif chemokine ligand 7; CXCL1/Cxcl1: C-X-C motif chemokine ligand 1; CXCL12/Cxcl12: C-X-C motif chemokine ligand 12; CXCL14: C-X-C motif chemokine ligand 14; COL1A1: collagen type I alpha 1 chain; COL1A2: collagen type I alpha 2 chain; COL3A1/Col3a1: collagen type III alpha 1 chain; COL6A3: collagen type VI alpha 3 chain; COL10A1: collagen type X alpha 1 chain; COL12A1: collagen type XII alpha 1 chain; COL14A1: collagen type XIV alpha 1 chain; CTHRC1: collagen triple helix repeat containing 1; C3: complement component 3; DLK1: delta like non-canonical notch ligand 1; Eb1: ErbB2 interacting protein; FAP/Fap: fibroblast activation protein alpha; FBN1/Fbln1: fibrillin 1; FN1: fibronectin 1; H2-Aa: histocompatibility 2, class II antigen A, alpha; H2-Ab1: histocompatibility 2, class II antigen A, beta 1; IL6: interleukin 6; LUM/Lum: lumican; Ly6c1: lymphocyte antigen 6 complex, locus C1; MME: membrane metalloendopeptidase; PDGFRA/Pdgfra: platelet derived growth factor receptor alpha; Pdgfa: platelet derived growth factor subunit A; RGS5: regulator of G protein signaling 5; SFRP1/Sfrp1: secreted frizzled related protein 1; SLPI/Slpi: secretory leukocyte peptidase inhibitor; SAA1: serum amyloid A1; Saa3: serum amyloid A3; Smoc2: SPARC related modular calcium binding 2; TGFB1/Tgfb1: transforming growth factor beta 1; Vcan: versican.

**Table 2:** Summary of the specific CAF subtypes and features in cancers

**CAF subtype**	**Cancer type**	**Model**	**Gene signature**	**Role in cancer**	**Ref.**
Metabolic state (meCAFs)	Pancreatic cancer	Human	*PLA2G2A*, *CRABP2*, *LDHB*, *PGK1*	Promote tumorigenesis via translation, glycolysis, noncanonical NF-κB signaling, P21/P27 degradation, and the MYC pathway	[33]
EMT-like CAF (eCAFs)	ICC	Human	*KRT19*, *KRT8*, *SAA1*	Promote tumorigenesis by EMT in TME	[36]
	Gastric cancer	Human	*KRT19*, *CLDN18*		[64]
Lipid-laden CAFs	Pancreatic cancer	Setd2-deficient KSC mouse model	*Abca8a*, *Pdpn*, *Fap*	Enhance tumor progression by providing lipids for mitochondrial oxidative phosphorylation via ABCA8a transport	[81]
Proliferative CAFs (pCAFs)	Gastric cancer	Human	*TOP2A*, *STMN1*, *MKI67*, *HIST1H4C*, *CENPF*	Promote various biological processes and immune responses	[65]
Vascular CAFs (vCAFs)	Breast, lung, colon, head-and-neck, pancreatic cancer	Human	*COL18A1*, *VEGF*, *MCAM*, *NOTCH3*	Promote the proliferation, survival, and metastasis of cancer cells through angiogenesis and vascularization	[27,28]
	Breast cancer	Mouse	*Col18a1*, *Epas1*, *Nr2f2*, *Notch3*, *Nidogen-2*		[66]
	Gastric cancer	Human	*MACM*, *MYLK*, *RGS5*		[61]
Developmental CAFs/dividing CAFs (dCAFs)	Breast, lung, colon, head-and-neck, pancreatic cancer	Human	*TUBA1B*, *MKI67*	Promote cell division and tissue morphogenesis	[28]
	Breast cancer	Mouse	*Scrg1*, *Sox9*, *Sox10*		[66]
Peripheral nerve-like CAFs (CAF_PN_)	Gastric, colorectal, ovarian, pancreatic, prostatic cancer	Human	*MPZ*, *S100B*, *LGI4*, *PLP1*, *SOX2*, *SOX10*	Promote perineural invasion and are associated with poor prognosis	[14]
Interferon-response CAFs (ifnCAFs)	Breast, lung, colorectal, head-and-neck	Human	*CXCL9*, *CXCL10*, *CXCL11*, *IDO1*, *IL-32*	Upregulate genes in response to interferons; however, it remains unclear whether they promote or inhibit tumor growth in cancer	[28]
	Pancreatic cancer	Human	*RSAD2*, *IFIT3*, *CXCL10*, *DDX58*	Inhibited the invasive potential of tumor cells and promoted an anti-tumor response in neutrophils associated with tumors *in vivo* and *in vitro*	[82]
		Mouse	*Cxcl10*, *Rsad2*, *Ifit3*		
Tumor-like CAFs (tCAFs)	Breast, lung, colon, head-and-neck, pancreatic cancer	Human	*CAIX*, *ENO1*, *GAPDH*, *MME*, *NDRG1*, *PDPN*, *TMEM158*	Promote tumor growth through angiogenesis	[28]
Reticular-like CAFs (rCAFs)	Breast, lung, colon, head-and-neck, pancreatic cancer	Human	*CCL19*, *CCL21*	Promote TLS formation and T cell homing	[28]
PDPN^+^ CAFs (pCAFs)	Breast, lung cancer	Human	*PDPN*, *FAP*	Promote tumor growth and metastasis by inhibiting T cell activation	[38,78]
	Breast cancer	Mouse	*Pdpn*, *Il-6*, *Cxcl12*, *Saa3*, *Acta2*, *Fbn1*, *Cxcl1*		[78]
S100A4^+^ CAFs (sCAFs)	Breast cancer	Mouse	*S100a4*, *Spp1*, *H2-Aa*, *Hspd1*, *CD74*, *Slpi*, *H2-Ab1*	Improve clinical outcomes by activating the immune system	[78]
Zinc-transport CAFs (zCAFs)	Lung cancer	Human	*ZIP1*, *S100A4*, *CX43*	Promote chemoresistance through transferring Zn^2+^ to neighbouring cancer cells via gap junctions	[83]
		Mouse	*Zip1*, *Notch2*, *S100a4*, *Spry2*, *Mt1/2*		
Meflin-positive CAFs	Pancreatic cancer	Human	*ISLR*, *PDGFRa*, *GLI1*	Be correlated with favorable patient outcome Enhance gemcitabine sensitivity	[84,85]
	Lung cancer	Human	*ISLR*, *PDGFRa*	Enhanced infiltration of CD4^+^ T cells and potentiate ICI response *in vivo*	[86]
CD105^neg^ CAFs	Pancreatic cancer	Mouse	*Cxcl2*, *Gas1*, *Bmp2*, *Nos2*	Support anti-tumor immunity to control tumor growth	[87]

CAF: Cancer-associated fibroblast; NF-κB: nuclear factor kappa-B; MYC: myelocytomatosis oncogene; EMT: epithelial-mesenchymal transition; ICC: intrahepatic cholangiocarcinoma; TME: tumor microenvironment; KSC: KrasG12D; Smad4flox/flox; Cdkn2aflox/flox mouse model of pancreatic ductal adenocarcinoma; ABCA8a: ATP binding cassette subfamily A member 8; TLS: tertiary lymphoid structure; PDPN: podoplanin; ICI: immune checkpoint inhibitor; ACTA2/Acta2: actin alpha 2, smooth muscle; Abca8a: ATP binding cassette subfamily A member 8a; Bmp2: bone morphogenetic protein 2; CCL19: C-C motif chemokine ligand 19; CCL21: C-C motif chemokine ligand 21; CXCL1/Cxcl1: C-X-C motif chemokine ligand 1; CXCL10/Cxcl10: C-X-C motif chemokine ligand 10; CXCL11: C-X-C motif chemokine ligand 11; CXCL12/Cxcl12: C-X-C motif chemokine ligand 12; Cxcl2: C-X-C motif chemokine ligand 2; CXCL9: C-X-C motif chemokine ligand 9; CAIX: carbonic anhydrase IX; CENPF: centromere protein F; CLDN18: claudin 18; COL18A1/Col18a1: collagen type XVIII alpha 1 chain; DDX58: DEAD-box helicase 58; TOP2A: DNA topoisomerase II alpha; ENO1: enolase 1; Epas1: endothelial PAS domain protein 1; FAP/Fap: fibroblast activation protein alpha; FBN1/Fbln1: fibrillin 1; CX43: gap junction protein alpha 1; GLI1: GLI family zinc finger 1; GAPDH: glyceraldehyde-3-phosphate dehydrogenase; Gas1: growth arrest specific 1; Hspd1: member 1; H2-Aa: histocompatibility 2, class II antigen A, alpha; H2-Ab1: histocompatibility 2, class II antigen A, beta 1; HIST1H4C: histone cluster 1 H4 family member C; ISLR: immunoglobulin superfamily containing leucine rich repeat; IDO1: indoleamine 2,3-dioxygenase 1; IFIT3/Ifit3: interferon induced protein with tetratricopeptide repeats 3; IL-32: interleukin 32; IL-6: interleukin 6; KRT19: keratin 19; KRT8: keratin 8; LDHB: lactate dehydrogenase B; LGI4: leucine rich repeat LGI family member 4; MCAM: melanoma cell adhesion molecule; MME: membrane metalloendopeptidase; Mt1/2: metallothionein 1/2; MYLK: myosin light chain kinase; MPZ: myelin protein zero; Nidogen-2: nidogen 2; NDRG1: N-Myc downstream regulated 1; Nos2: nitric oxide synthase 2, inducible; Notch2: notch receptor 2; NOTCH3/Notch3: notch receptor 3; PGK1: phosphoglycerate kinase 1; PLA2G2A: phospholipase A2 group IIA; PDGFRA/Pdgfra: platelet derived growth factor receptor alpha; PDPN/Pdpn: podoplanin; PLP1: proteolipid protein 1; RSAD2/Rsad2: radical S-adenosyl methionine domain containing 2; RGS5: regulator of G protein signaling 5; S100A4: S100 calcium binding protein A4; S100a4: S100 calcium binding protein A4; S100B: S100 calcium binding protein B; Spp1: secreted phosphoprotein 1; SLPI/Slpi: secretory leukocyte peptidase inhibitor; SAA1: serum amyloid A1; Saa3: serum amyloid A3; SOX10/Sox10: SRY-Box transcription factor 10; SOX2: SRY-Box transcription factor 2; Sox9: SRY-Box transcription factor 9; Spry2: sprouty RTK signaling antagonist 2; STMN1: stathmin 1; Scrg1: stimulating retinoic acid gene 1; TMEM158: transmembrane protein 158; TUBA1B: tubulin alpha 1b; VEGF: vascular endothelial growth factor A; ZIP1/Zip1: zrt and irt like protein 1.

## MOLECULAR CROSSTALK BETWEEN CSCS AND CAFS MEDIATING DRUG RESISTANCE

### CSCs activate CAFs in TME

**Figure 1:**
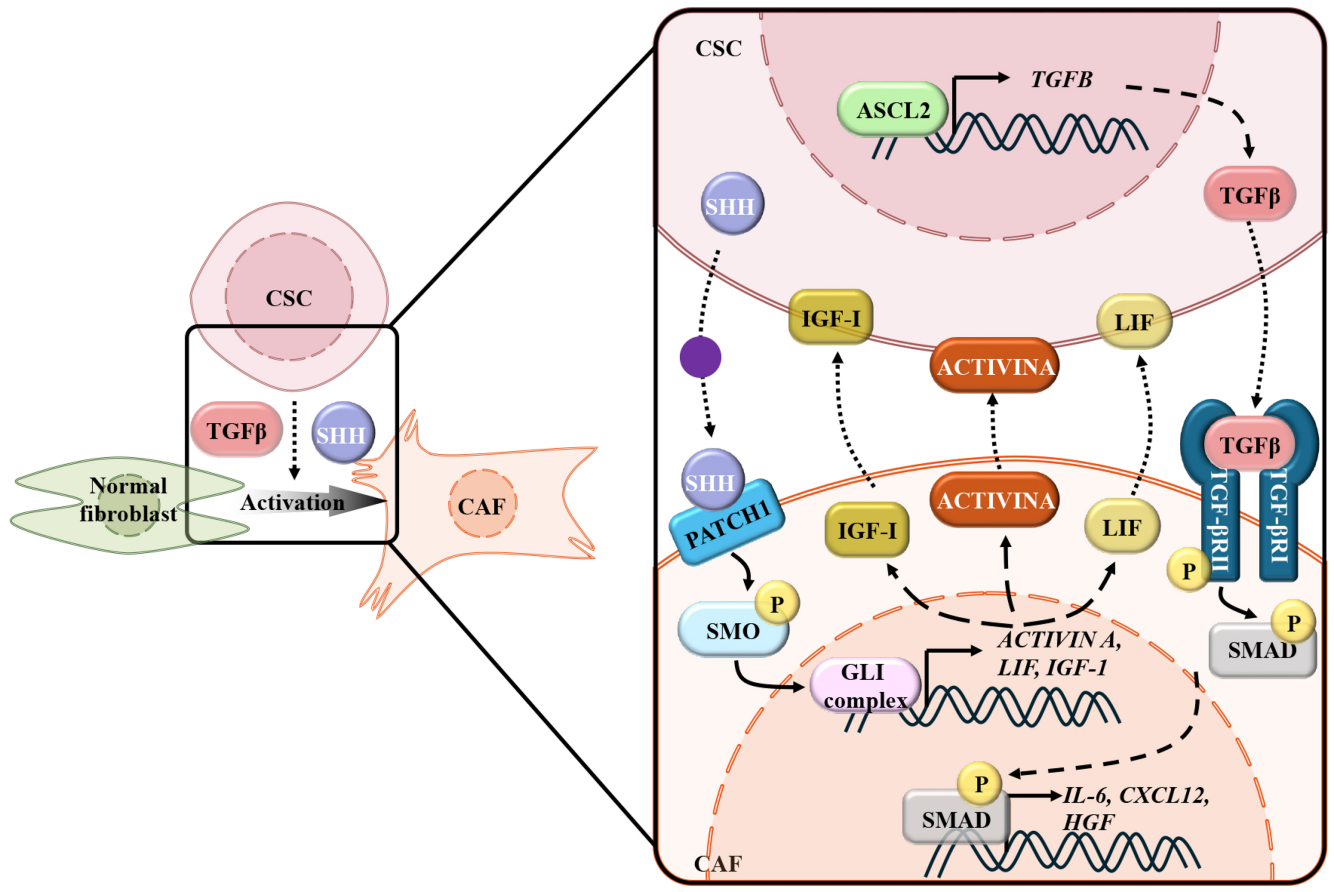
CSCs promote the activation of CAFs. Elevated ASCL2 in CSCs promotes the transcription and secretion of TGF-β, inducing the transcription of IL-6, CXCL12, and HGF, as well as the transformation of CAFs from normal fibroblasts. CSCs-derived SHH binds to PATCH1 on the CAFs and subsequently activates glutaminase (GLI) complex-mediated transcription of ACTIVIN A, LIF, and IGF1, which reciprocally maintain the stemness of CSCs. CSCs: Cancer stem cells; CAFs: cancer-associated fibroblasts; ASCL2: achaete-scute family BHLH transcription factor 2; TGF-β: transforming growth factor-β; IL-6: interleukin-6; CXCL12: chemokine (C-X-C motif) ligand 12; HGF: hepatocyte growth factor; SHH: Sonic Hedgehog; PATCH1: patched homolog 1; GLI: GLI family zinc finger; LIF: leukemia inhibitory factor; IGF1: insulin-like growth factor 1; SMO: smoothened; SMAD: mothers against decapentaplegic Homolog.

CSCs activate CAFs through multifaceted secretory programs, establishing a self-reinforcing TME that drives malignancy and therapy resistance. They secrete a repertoire of soluble factors (TGF-β1 and EVs) which recruit and reprogram resident fibroblasts into CAFs^[10,11,88,89]^. These CSC-derived signals sustain CSC stemness and therapeutic escape, contributing to tumor recurrence and drug resistance [Figure 1].

In microsatellite-stable CRC, achaete-scute family BHLH transcription factor 2 (ASCL2)-upregulation elevates CSC markers and TGF-β1 secretion via direct binding to the transforming growth factor beta (*TGFB*) promoter. ASCL2-knockdown attenuates CAFs activation, lowering α-SMA expression and pro-tumorigenic cytokines (IL-6, HGF, and CXCL12)^[88]^. The ASCL2 upregulation by promoter demethylation promotes 5-fluorouracil (5-FU) resistance in gastric cancer cells^[90]^ and CAFs-mediated 5-FU resistance by the TGF-β1 signaling pathway in esophageal squamous cell carcinoma^[91]^. So, we hypothesize that ASCL2-driven TGF-β1 signaling in microsatellite-stable colorectal cancer coordinately induces CAF-mediated therapeutic resistance through a conserved mechanism. In TNBC, CSCs activate IRF6 expression in fibroblasts, driving CAFs transformation and aberrant collagen/fibronectin deposition for pro-tumorigenic niches^[12]^. Research in basal-like mammary tumors further uncovers the mechanism of the bidirectional signaling axis between CSCs-CAFs. It was reported that CD24^+^CD49f^hi^ CSCs localize at the tumor-stroma interface adjacent to vimentin^+^ CAFs. Transcriptional profiling and gene set enrichment analysis identified Hedgehog pathway activation in CAFs, despite the absence of Hedgehog ligands in these cells. Instead, CSCs were observed to produce Sonic Hedgehog (SHH), which activated paracrine Hedgehog signaling in neighboring CAFs, promoting their proliferation and inducing the secretion of ACTIVIN A, insulin-like growth factor 1 (IGF-1), and leukemia inhibitory factor (LIF). These CAF-derived factors, in turn, reinforced CSC self-renewal, establishing a bidirectional signaling axis^[92]^.

EVs have emerged as critical mediators of CSC-CAF crosstalk. EVs derived from Piwil2-induced CSCs reprogram normal fibroblasts into CAFs *in vitro*^[10]^*.* In the TNBC mouse model, CSC-EVs were reported to activate mouse CAFs, which enhances pre-metastatic niche formation and angiogenesis in the lung^[11]^. Further study in oral squamous cell carcinoma (OSCC) reported that CSC-EVs enriched in miR-21-5p promote CAF activation and cisplatin resistance via TGF-β1^[89]^. In colon cancer, CSC-derived exosomes activate CAFs and promote 5-FU resistance through miRNA-1246 and IL-6/STAT3/β-catenin signaling^[9]^. These findings underscore the versatility of EVs in propagating CSC-driven stromal reprogramming and drug resistance.

Activated CAFs reciprocally amplify CSC aggressiveness by releasing IL-6/IL-8 and matrix metalloproteinases, which promote tumor stemness^[11]^. This bidirectional feedback loop creates a vicious cycle, perpetuating a therapy-resistant TME.

### CAFs fuel CSCs in TME

CAFs directly engage with CSCs through surface receptor-ligand interactions, reinforcing stemness. Early studies by Kinugasa *et al.* revealed that CD44^+^ CAFs in CRC hypovascular niches sustain CSC properties and confer resistance to 5-FU, although the precise molecular mediators remained^[93]^. Ovarian cancer studies further illustrate adjacency-dependent signaling. Co-culture of ovarian cancer cell line 3 (OVCAR3) cells with primary ovarian CAFs enriches ALDH^+^ CSCs, particularly at CAF-tumor interfaces. A pivotal mechanism involves Notch and Wnt signaling, which balances the self-renewal and differentiation of CSCs^[94]^. Yet, the findings on the direct crosstalk between CSCs and CAFs remain limited. In this section, we will outline recent advances in mechanisms underlying CAF-mediated stemness maintenance through multidimensional aspects, encompassing biochemical signaling, structural remodeling, and metabolic symbiosis.

#### ECM remodeling by CAFs determines CSC fate, promoting therapeutic resistance

**Figure 2:**
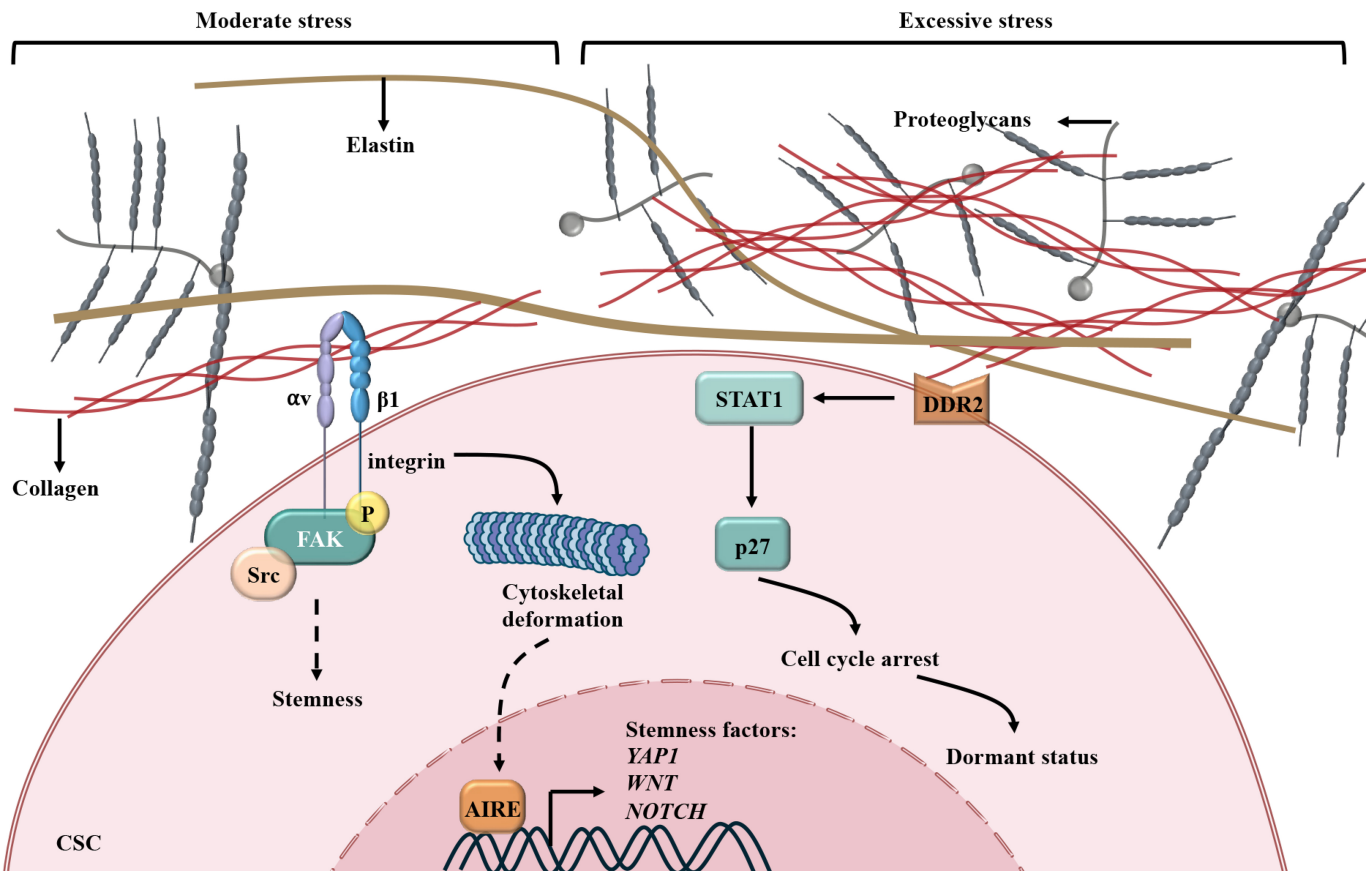
CAFs create a biomechanical niche for CSCs, mediating resistance. Integrins interact with collagen produced by CAFs and activate FAK-Src signaling or initiate cytoskeletal deformation and subsequent AIRE-mediated transcription of stemness factors, enhancing the stemness of CSCs. When the ECM becomes stiffer and exerts excessive stress on CSCs, DDR2 on CSCs receives the biomechanical signal and causes cell cycle arrest via the STAT1/P27 axis, facilitating CSCs into a dormant status. CAFs: Cancer-associated fibroblasts; CSCs: cancer stem cells; FAK: focal adhesion kinase; AIRE: autoimmune regulator gene; ECM: extracellular matrix; DDR2: discoidin domain receptor 2; STAT1: signal transducer and activator of transcription 1; YAP1: Yes1-associated transcriptional regulator 1; WNT: Wnt family member; NOTCH: neurogenic locus notch homolog protein.

The CSC niche is a dynamic ecosystem shaped by cellular interactions and ECM remodeling, where tumor-associated macrophages, mesenchymal stem cells, endothelial cells, and CAFs collaborate to sustain stemness^[95]^. Within this niche, CAFs act as central architects, orchestrating biochemical and biomechanical factors through ECM components, including collagens, fibronectin, and hyaluronic acid. These elements provide structural support and activate signaling networks critical for CSC self-renewal, survival, and therapy resistance^[96]^ [Figure 2].

Collagen-rich microenvironments play a defining role in CSC stemness maintenance and chemotherapy resistance. In PDAC, 3D-collagen I promotes gemcitabine (GEM) resistance through membrane type 1 matrix metalloproteinase (MT1-MMP)-mediated expression of high mobility group A2 (HMGA2)^[97]^. Additionally, type I collagen engages β1-integrin on tumor cells, triggering FAK phosphorylation at tyrosine 397. This activation amplifies clonogenicity and tumor-initiating capacity, as evidenced by β1-integrin ablation suppressing colony formation and FAK overexpression expanding the ALDH^+^ CSC population. These findings highlight collagen-mediated FAK signaling as a crucial component of CSC-driven tumorigenesis^[98]^.

Beyond biochemical signaling, ECM biomechanics affect the transition of CSCs from stemness to quiescence. Breast cancer cells cultured in 3D-Matrigel with a mechanical force of approximately 45 Pa derived from integrin β1/3 receptors exhibit enhanced stemness, which is regulated by the cytoskeleton-autoimmune regulator gene (AIRE) axis. Excessive rigidity (450 Pa) turns CSCs into quiescent status via discoidin domain receptor 2 (DDR2)/signal transducer and activator of transcription 1 (STAT1)/P27 signaling^[99]^.

These studies indicate that selective ECM deposition and remodeling can balance stemness, proliferation, and dormancy via biochemical interaction and niche mechanics. This suggests the therapeutic potential of targeting stromal-ECM crosstalk to disrupt CSC resilience in aggressive malignancies.

#### CAFs maintain stemness via cytokines, chemokines, and others in drug resistance

**Figure 3:**
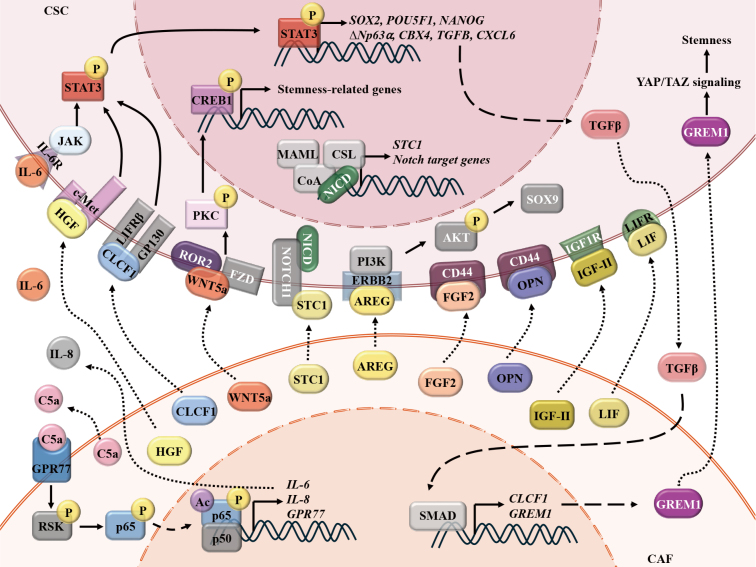
CAFs maintain the stemness of CSCs via secreted proteins. CAFs secrete a panel of proteins, including HGF, IL-6, IL-8, CLCF1, WNT5A, STC1, AREG, FGF2, OPN, IGF-II, and LIF, which can be recognized by corresponding receptors on the CSCs and subsequently maintain the stemness of CSCs via the STAT3, PI3K/AKT, NOTCH, and other pathways. Cancer cells-derived TGF-β promotes the activation of CAFs and the secretion of DAN family BMP antagonist (GREM1), which, in turn, activates YAP/TAZ pathways in CSCs. CAFs: Cancer-associated fibroblasts; CSCs: cancer stem cells; HGF: hepatocyte growth factor; IL-6: interleukin-6; IL-8: interleukin-8; CLCF1: cardiotrophin-like cytokine factor 1; WNT5A: Wnt family member 5A; STC1: stanniocalcin-1; AREG: amphiregulin; FGF2: fibroblast growth factor-2; OPN: osteopontin; IGF-II: insulin-like growth factor 2; LIF: leukemia inhibitory factor; STAT3: signal transducer and activator of transcription 3; PI3K/AKT: phosphatidylinositol 3-kinase/protein kinase B; NOTCH: neurogenic locus notch homolog protein; TGF-β: transforming growth factor-β; DAN: deoxyribonucleic acid; BMP: bone morphogenetic protein; GREM1: gremlin-1; YAP/TAZ: Yes1-associated transcriptional regulator/transcriptional co-activator with PDZ-binding motif; SOX2/9: SRY (sex determining region Y)-box 2/9; POU5F1: POU class 5 homeobox 1, also known as octamer-binding transcription factor 4 (OCT4); NANOG: Nanog homeobox; CBX4: chromobox homolog 4; CXCL6: chemokine (C-X-C motif) ligand 6; CREB1: CAMP responsive element binding protein 1; MAML: Mastermind-like; CSL: C-promoter binding factor 1, Suppressor of Hairless/Lin-12, and Glp-1 phenotype; NICD: Notch intracellular domain; JAK: janus kinase; LIFRβ: leukemia inhibitory factor receptor β; ROR2: receptor tyrosine kinase-like orphan receptor 2; FZD: Frizzled; ERBB2: erb-b2 receptor tyrosine kinase 2; GPR77: G protein-coupled receptor 77; RSK: ribosomal S6 kinase; SMAD: mothers against decapentaplegic homolog.

CAFs sustain CSC stemness through paracrine signaling, deploying a diverse arsenal of cytokines, growth factors, and signaling modulators to sculpt a permissive niche [Figure 3]. IL-6 and WNT5A emerge as linchpins across malignancies.

In breast and lung cancers, complement component 5a (C5a) receptor-G protein-coupled receptor 77 (GPR77) mediates NF-κB signaling, maintains CD10^+^GPR77^+^ CAF activation, and induces IL-6/IL-8 production that fosters CSC self-renewal, which promotes docetaxel or cisplatin chemoresistance^[67]^. In HCC, CAF-derived HGF and IL-6 activate receptor tyrosine kinase, proto-oncogene (c-Met) and IL-6R receptors on CD24^+^ CSCs, inducing STAT3-phosphorylation at Tyr705, upregulating stemness regulators SRY-box transcription factor 2 (SOX2)/octamer-binding transcription factor 4 (OCT4)/Nanog homeobox (NANOG), and increasing the Sorafenib resistance^[100]^. Similarly, CAF-secreted IL-6 triggers JAK/STAT3 signaling in squamous cell carcinomas, elevating the CSC marker delta N-terminal truncated isoform of tumor protein p63, alpha isoform (ΔNp63α) and chromatin-modifying protein chromobox homolog 4 (CBX4). CBX4 inhibition disrupts spheroid formation, highlighting its necessity in CSC phenotype maintenance^[101]^.

ICAM1^+^ iCAFs secrete fibroblast growth factor-2 (FGF2) in recurrent bladder cancer, engaging CD44 to sustain stemness^[102]^. scRNA-seq of HGSOC reveals CAFs as dominant sources of Wnt5a, a non-canonical Wnt ligand, and promoting carboplatin resistance. Unlike canonical Wnt signaling, which operates within cancer cells, CAFs-derived Wnt5a activates non-canonical pathways, including protein kinase C (PKC) and CAMP responsive element binding protein 1 (CREB1), in adjacent CSCs via receptor tyrosine kinase-like orphan receptor 2 (ROR2). ROR2-knockdown suppresses ALDH activity, while Wnt5a inhibitors synergize with carboplatin in preclinical models, underscoring therapeutic potential^[94]^.

Other CAF-secreted proteins include insulin-like growth factor 2 (IGF-II), LIF, osteopontin (OPN), and stanniocalcin-1 (STC1)^[103-106]^. Lung CAFs secrete IGF-II, which binds insulin-like growth factor 1 receptor (IGF1R) on CSCs to activate NANOG/SOX2, sustaining stem cell plasticity and increasing resistance to various chemo-agents, including etoposide, docetaxel, vinorelbine detartrate, and cisplatin^[103]^. In BC, CAF-derived LIF activates leukemia inhibitory factor receptor (LIFR) on cancer cells, driving NANOG/OCT4 expression while expanding the CD44^+^/CD24^-^ CSC population. LIFR-blocking abolishes this effect, revealing its indispensability in CSC-dedifferentiation^[104]^. Circular RNA circFARP1 (hsa_circ_0002557) enables pancreatic CAFs to promote GEM resistance through the LIF/STAT3 axis^[107]^. So, in the future, we should focus on delineating the mechanistic underpinnings of CAF-derived LIF-driven chemoresistance in BC. In PC, CAFs-CM upregulate CSC markers and drug resistance genes *in vitro* and enhance tumorigenicity *in vivo*. Proteomic profiling identified OPN as a key ligand persistently elevated in CAFs-CM, with co-localization of OPN-CD44 in tumor tissues. Silencing CD44 or OPN abolishes tumor sphere-formation and stemness, confirming the OPN-CD44 axis as a critical driver of CAF-CSC crosstalk^[105]^. HCC CAF-derived STC1 binds to Notch1 receptor on neighboring-tumor cells, which induces Notch1 proteolytic cleavage and Notch intracellular domain (NICD) release. NICD translocates to the nucleus and complexes with the transcription factor CSL, which binds to the STC1 promoter. The NICD-CSL interaction establishes a self-amplifying transcriptional loop, driving persistent STC1 overexpression, sustained Notch pathway activation, HCC stemness, and promoting sorafenib resistance^[106]^. Moreover, scRNA-seq analysis demonstrated frequent interactions between iCAFs and CSCs via Wnt and EGF signaling pathways. Intercellular crosstalk analysis further revealed that the ligand-receptor pair AREG-EGFR/ERBB2 was significantly enriched in iCAFs-CSCs communication. Spatial transcriptomic results confirmed the co-localization and positive correlation between iCAFs and CSCs in both early and advanced gastric cancer, with spatially consistent expression patterns of AREG and ERBB2. Mechanistically, iCAFs-secreted AREG binds to ERBB2 receptor on tumor cells, activating downstream protein kinase B (AKT) phosphorylation and upregulating the transcription factor SOX9. The AREG-ERBB2 axis reinforces CSCs’ self-renewal tumor stemness and carboplatin and cisplatin resistance^[61]^.

Feedback loops amplify the interplay between CAFs-CSCs. In HCC, CAF-derived cardiotrophin-like cytokine factor 1 (CLCF1) activates extracellular signal-regulated kinase 1/2 (ERK1/2)-STAT3 signaling, upregulating SOX2/NANOG/cellular Myelocytomatosis oncogene (c-MYC) while stimulating TGF-β and CXCL6 production in cancer cells. These factors reciprocally enhance CLCF1 secretion by CAFs, creating a self-reinforcing circuit^[108]^. In BC, cancer cell-derived TGF-β and pro-inflammatory cytokines induce CAFs to secrete the bone morphogenetic protein (BMP) antagonist-GREM1, which blocks BMP/SMAD signaling in cancer cells. This inhibition enhances Yes1-associated transcriptional regulator/transcriptional co-activator with PDZ-binding motif (YAP/TAZ) and pluripotency factors, facilitating CSC expansion^[109]^. However, in colorectal cancer, the αSMA^+^ CAF subpopulation exerts a tumor-suppressive effect by secreting bone morphogenetic protein 4 (BMP4) to inhibit the stemness of leucine-rich repeat-containing G-protein coupled receptor 5 (Lgr5)^+^ CSCs, indicating that the regulation of CSCs by the CAF subpopulation is bidirectional (promoting or inhibiting) rather than a single mode^[110]^. In esophageal squamous cell carcinoma, tumor-derived IL-1α induces CAFs senescence via nuclear factor erythroid 2-related factor 2 (NRF2), triggering IGF1 secretion via nuclear factor I A (NFIA). The secreted IGF1 suppresses AMP-activated protein kinase (AMPK) in CSCs, reinforcing stemness and chemotherapy resistance^[111]^.

Collectively, these studies illuminate the specific CAF subpopulation as master regulators of CSC stemness and drug resistance, exploiting paracrine networks to hijack developmental pathways, suppress differentiation signals, and orchestrate metabolic and epigenetic reprogramming.

#### CAFs-derived EV-mediated signal communication in the regulation of drug resistance

CAFs regulate CSC plasticity through EV-mediated transfer of bioinformation, especially non-coding RNAs, orchestrating oncogenic signaling cascades. Donnarumma et al. identified CAF-derived exosomes enriched in miR-21-5p/miR-378e/miR-143-3p as key enhancers of breast cancer stemness, though their precise mechanistic interplay remains unknown^[112]^. In CRC, CAF-derived miR-92a-3p promotes tumor stemness and 5-FU/oxaliplatin (L-OHP) resistance by targeting F-box and WD repeat domain containing (FBXW7) and modulator of apoptosis 1 (MOAP1) in cancer cells^[113]^. In bladder cancer, CAF-secreted miR-146a-5p promotes stemness and chemoresistance by dual mechanisms. First, miR-146a-5p enhances sushi, von Willebrand factor type A, EGF, and pentraxin domain-containing 1 (SVEP1)-mediated tumor-stromal adhesion by recruiting transcription factor YY1 to the *SVEP1* gene promoter. Second, miR-146-5a targets AT-rich interaction domain 1A (ARID1A) and AMP-activated catalytic subunit alpha 2 (PRKAA2, also known as AMPKα2) to activate mTOR/STAT3 pathways. This dual signaling fosters CSC expansion and GEM resistance^[114]^. PDAC CAFs employ circular RNA circFARP1 (hsa_circ_0002557) to stabilize caveolin-1 (CAV1) by blocking its ubiquitination by zinc and ring finger 1 (ZNRF1), thereby enhancing LIF secretion. LIF derived from CAFs upregulates SOX2, which promotes the survival of CSCs and confers resistance to GEM. Neutralizing LIF abolishes these effects, underscoring its central role in maintaining stemness and mediating GEM resistance^[107]^.

Wnt pathway activation as a conserved mechanism of CAFs-EV-driven stemness. In HCC, CAFs-derived miR-92a-3p suppresses axis inhibition protein 1 (AXIN1), a negative regulator of Wnt/β-catenin, leading to nuclear β-catenin accumulation and CSC enrichment^[115]^. Renal cell carcinoma CAFs deploy miR-181d-5p to suppress ring finger protein 43 (RNF43)-an E3 ubiquitin ligase that degrades Frizzled receptors, resulting in Wnt/β-catenin hyperactivation. The Cancer Genome Atlas (TCGA) data corroborate Wnt pathway enrichment in high-CAF tumors, with RNF43-knockdown elevating β-catenin/OCT4/aldehyde dehydrogenase 1 family member A1 (ALDH1A1)^[116]^. Another study in CRC identified CAFs-secreted exosomal long non-coding RNA H19 as a competing endogenous RNA, sponging miR-141 to activate Wnt/β-catenin signaling. This axis stabilizes β-catenin, upregulating downstream targets c-MYC, cyclin D1 (CCND1), and CD44, amplifying CSC traits and oxaliplatin resistance^[117]^.

Collectively, these studies illuminate CAF-EVs as molecular couriers that hijack non-coding RNAs to rewire CSC signaling networks. By targeting tumor-suppressive miRNAs, CAFs amplify oncogenic pathways such as Wnt/β-catenin/STAT3/mTOR, cementing their role as architects of therapy-resistant niches.

#### Role of metabolic symbiosis in drug resistance between CAFs and CSCs

Michael Lisanti’s “Reverse Warburg effect” revealed CAFs as glycolytic hubs that produce lactate, which adjacent tumor cells utilize via monocarboxylate transporter 1/4 (MCT1/4) to fuel oxidative phosphorylation (OXPHOS), thereby formalizing stromal-tumor metabolic symbiosis. CAFs additionally provide glutamine, ketone bodies, and fatty acids to sustain tumor OXPHOS. Under nutrient stress, ovarian CAFs utilize any nitrogen and carbon source to synthesize glutamine for cancer cells. In return, cancer cells provide glutamate and lactate for glutamine synthesis in CAFs^[118]^. Additionally, exosomes function as critical nutrient vectors to transfer amino acids/tricarboxylic acid (TCA) cycle intermediates from pancreatic CAFs to cancer cells^[119]^. Autophagy in CAFs further enables nutrient flexibility. Lung CAFs provide dipeptides, while pancreatic CAFs secrete alanine, reducing tumor reliance on glucose and glutamine^[120]^.

Recent evidence suggests that CAFs drive CSC traits through metabolite-mediated metabolic reprogramming. CD10 of CD10^+^GPR77^+^ CAFs degrade osteogenic growth peptide (OGP) in BC, suppressing its C-terminal bioactive domain [YGFGG (a pentapeptide sequence of tyrosine-glycine-phenylalanine-glycine-glycine)] and sustaining docetaxel chemoresistance. OGP inhibition elevates stearoyl-Coenzyme A (CoA) desaturase-1 (SCD1), a lipid desaturase critical for CSC proliferation, via NF-κB activation. SCD1 increases monounsaturated fatty acids (e.g., C16:1/C16:0), essential for mammosphere formation. CD10-knockdown abolishes this effect, linking lipid metabolism to stemness^[121]^. Tetraspanin-8 (TSPAN8) in myCAFs recruits MAPK11 to phosphorylate retinoblastoma binding protein 6 (RBBP6), stabilizing sirtuin 6 (SIRT6) and inducing senescence. Proximity to aldehyde dehydrogenase 1 (ALDH1)^+^ CSCs highlights spatial metabolic coupling. TSPAN8^+^ CAFs secrete aspartate and proline modulated by glutaminase (GLS1) and pyrroline-5-carboxylate reductase 1 (PYCR1) via the SIRT6-MYC axis, fueling CSC metabolic demands and chemoresistance^[122]^. In cholangiocarcinoma (CCA), TGF-β-induced CAFs express lysyl oxidase (LOX), which enhances mitochondrial OXPHOS via upregulation of mitochondrial transcription factor A (TFAM), reinforcing CSC stemness^[123]^. In OSCC, CAF-derived lactate upregulates discs large homolog 5 (DLG5), stabilizing macrophage stimulating 1 (MST1) to activate Hippo signaling via Yes-associated protein 1 (YAP1)-phosphorylation, promoting CSC traits^[8]^.

Targeting the metabolic symbiosis between CAFs and CSCs using inhibitors of lactate transporters, autophagy, SCD1, and PYCR1 could disrupt stemness and overcome resistance. A synergistic blockade of the Hippo/NF-κB/MYC networks holds therapeutic potential. This framework elucidates CAFs-driven metabolic support for CSCs, revealing vulnerabilities in the TME.

In summary, the above findings demonstrate that crosstalk between CAFs and CSCs is widely applicable in drug resistance across solid tumors. The specific CAF subpopulation regulates CSC stemness through multiple mechanisms, including the secretion of various factors and exosomes, ECM remodeling, and metabolic symbiosis, thereby promoting drug resistance. This has been reported in various cancers, such as breast, lung, colorectal, gastric cancer, CCA, HCC, bladder cancer, and pancreatic cancer.

## TARGETING CAFS-CSCS CROSSTALK

Conventional therapies typically target aberrantly proliferating tumor cells but often spare quiescent CSCs, key mediators of therapeutic resistance. Significant efforts have been made to target CSCs through their markers or stemness-related pathways. However, advancing CSC-targeting agents to clinical maturity requires further preclinical validation and clinical trials^[7,124]^. However, these CSCs are additionally shielded by a specific component of CAFs-derived ECM, such as hyaluronic acid and collagen. This dense stromal barrier restricts drug penetration and immune infiltration, fostering a protective niche. To overcome these challenges, we will explore the following three strategies aimed at disrupting the CAFs–CSCs crosstalk: Targeting specific biomarkers associated with CAFs and CSCs; Employing small-molecule inhibitors against critical signaling pathways; Utilizing natural compounds that effectively target the interactive network between CAFs and CSCs.

### Targeting specific markers

Therapeutic strategies targeting CAFs have developed substantially since the pioneering use of FAP-directed monoclonal antibody in metastatic colon cancer; however, both Sibrotuzumab and Talabostat failed to progress beyond phase II clinical trials^[125,126]^. Instantly, one of the quinoline-based FAP inhibitors (FAPIs ^68^Ga-FAPI-04) developed by University Hospital Heidelberg outperformed ^18^F-fluorodeoxyglucose (FDG) in detecting cancerous lesions in 48 breast cancer patients^[127]^. However, FAPIs suffer from poor tumor retention. To overcome this, ^68^Ga-FAP-2286 was developed and showed promising imaging in preclinical models beyond breast cancer^[128,129]^. On the other hand, contradictory findings suggest that FAP, α-SMA, PDGFRα/β, and PDPN have dual roles; while they are often tumor-promoting, they can also inhibit tumors. Higher FAP levels in invasive breast ductal carcinoma have been correlated with better survival^[130]^. Depleting α-SMA^+^ myCAFs in a transgenic PDAC mouse model promoted tumor invasion, EMT, stem-like traits, and CD4^+^Foxp3^+^ Treg cell infiltration, while reducing OS^[29]^. The contradictory findings in preclinical murine models currently hinder the progression of α-SMA-targeted research toward clinical application. High PDGFRα expression in PDAC CAFs correlated with enhanced immune infiltration, robust T cell cytotoxicity, and improved survival^[131]^. PDPN^+^ CAFs were associated with longer disease-free survival (DFS) in CRC patients^[132]^, and their anti-tumor effect was mediated by the suppression of SCLC cell proliferation in a co-culture assay^[133]^. The heterogeneity of those CAFs may explain prior clinical trial failures. CAFs-targeted strategies have evolved from early FAP-directed antibodies to precision approaches that disrupt CAFs reprogramming pathways in various preclinical studies and clinical trials, which have been well reviewed elsewhere^[23,24,134]^. Despite significant progress in preclinical research, several therapeutic approaches, such as those targeting SHH-smoothened (SMO) or hyaluronic acid, have proven ineffective, sometimes even reducing patient survival^[135-138]^.

Targeting CAF-specific markers faces significant challenges in clinical translation. While factors such as FAP, α-SMA, PDGFRα/β, and PDPN have been explored as therapeutic targets, three major obstacles remain. First, the complex and evolving communication networks of CAFs contribute to substantial heterogeneity. Second, there is dynamic plasticity between pro-tumor and anti-tumor CAF subpopulations. Third, commonly used markers such as FAP, α-SMA, PDGFRα/β, and PDPN are not exclusive to CAFs, and the absence of truly specific biomarkers increases the risk of off-target effects. Crucially, definitive identifiers are still lacking for CAF subpopulations that specifically maintain CSCs and drive therapy resistance.

Therefore, disrupting the crosstalk between CAFs and CSCs represents a promising alternative strategy to overcome drug resistance. The current bottleneck lies in the absence of reliable markers for CAF subsets that exclusively promote CSC function and resistance. Future therapeutic efforts should shift from targeting individual multifunctional markers toward mapping and inhibiting precise signaling nodes within the CAF–CSC crosstalk network that are directly responsible for treatment-resistant phenotypes.

### Small-molecule inhibitors targeting specific signaling pathways

Several signaling pathways, including IL-6/STAT3, TGF-β, HGF/c-MET, Wnt/β-catenin, and Hedgehog signaling, are reported to be critical axes in CAFs-CSCs crosstalk. The potential strategies targeting these signaling pathways for overcoming stromal-CSCs-driven drug resistance have been reviewed^[139]^. Despite promising preclinical results, clinical translation has proven challenging. For instance, the anti-TGF-β monoclonal antibody SAR439459 showed synergy with PD-1 blockade and enhanced anti-tumor immunity in preclinical studies^[140]^. However, a subsequent clinical trial was terminated after the drug demonstrated a lack of efficacy and a significant bleeding risk in cancer patients (NCT04729725, clinicaltrials.gov). M7824 is a bifunctional molecule that targets TGF-β by fusing the transforming growth factor-beta receptor type II (TGF-βRII) receptor to an anti-programmed death-ligand 1 immunoglobulin G1 (PD-L1 IgG1) antibody. It has demonstrated anti-tumor efficacy in preclinical studies of a mouse model^[141]^. However, the phase III clinical trial was discontinued because it did not demonstrate superior efficacy relative to pembrolizumab (NCT04297748, clinicaltrials.gov)^[142]^. Similarly, the Hedgehog inhibitors, and GDC-0449 (vismodegib), exhibited anti-tumor activities in preclinical studies^[143,144]^. In a 42-month II trial, sonidegib showed robust efficacy and continuous tolerability in patients with advanced basal cell carcinoma (NCT01327053, clinicaltrials.gov)^[145]^. Unfortunately, a pilot clinical trial combining vismodegib with gemcitabine in metastatic pancreatic adenocarcinoma showed acceptable safety without new toxicities but failed to improve median progress free survival (PFS) or OS (NCT01088815, clinicaltrials.gov)^[146]^. Rilotumumab, an agent that inhibits c-MET (the c-MET receptor tyrosine kinase has been revealed as a target in various cancers), also failed to meet the primary endpoint and was associated with worse OS in a phase III clinical trial (NCT01697072, clinicaltrials.gov)^[147]^. Additionally, Runt-related transcription factor 1 (RUNX1) appears to be a novel target in CAFs. The RUNX1-specific inhibitor Ro5-3335 inhibits CAFs activation in a mouse model of Lewis lung carcinoma, suggesting therapeutic potential for RUNX1 inhibitors in attenuating pro-TME^[20]^. In colorectal cancer, CAF-derived lactate induces K453 lactylation of anthrax toxin receptor 1 (ANTXR1). Mechanistically, lactylation stabilizes ANTXR1, promoting cancer stemness and oxaliplatin chemoresistance through the Ras homolog family member C (RhoC)/rho-associated coiled-coil containing protein kinase 1 (ROCK1)/SMAD family member 5 (SMAD5) pathway. The MCT1/4 inhibitor 7-amino-5-chloro-8-quinolinecarboxamide (7ACC1) blocks CAF-tumor lactate shuttle, improving chemotherapy efficacy in cell-based xenografts and patient-derived xenografts models^[148]^. So far, the use of 7ACC1 has been limited to animal experiments, and no 7ACC1 reagents have moved into the clinic. Vitamin D was also shown to deactivate CAFs and reduce the secretion of tumor-promoting factors^[149,150]^. However, a phase II trial (NCT01516216, clinicaltrials.gov) investigating its supplementation as an adjunct to chemotherapy found no clinical benefit in CRC patients^[151]^.

Although preclinical models highlight the therapeutic potential of disrupting these networks, clinical outcomes emphasize the complexity and context-dependent nature of these targets. Future efforts may require combination strategies or patient stratification based on stromal biomarkers.

### Natural compounds targeting the CAFs–CSCs crosstalk

In addition to small-molecule inhibitors and targeting antibodies, certain natural compounds demonstrate dual efficacy by simultaneously disrupting CAFs and CSCs. Lee *et al.* established a TME-based drug screening platform to systematically identify natural compounds with multimodal activity against bulk cancer cells, CAFs, and CSCs. This platform highlighted that digoxin, a cardiac glycoside extracted from the foxglove plant, exhibits dual inhibitory effects on both CSC subpopulation and CAF-derived cytokine secretion at a clinically relevant dose of 1 nM^[152]^. In the phase I EDALINE trial (NCT02027376, clinicaltrials.gov), 3 of 12 metastatic TNBC patients showed clinical benefit from the combination therapy of smoothened inhibitors (sonidegib) and docetaxel, which targets breast CSCs and CAFs, including one complete response^[153]^. This platform also found berberine (BBR), a natural compound derived from various plants, as a potent anti-CAF inhibitor. While their scRNA-seq data confirm that dual targeting of CSCs and stromal cells inhibits the CSC subpopulation, the detailed mechanisms behind this interplay, particularly between CSCs and CAFs, remain to be explored to uncover new therapeutic vulnerabilities. Accumulating studies have reported the anti-tumor function of BBR^[154]^. BBR inhibits collagen synthesis and cytokine secretion of cardiac fibroblasts through the AMPK-mTOR-p70 ribosomal protein S6 kinase (p70S6K) signaling pathway^[155]^, CAF activation, ECM deposition^[156,157]^, and stemness through elevating the expression of miR-145 and miR-34a and decreasing IL-6 expression^[157]^. The efficacy of first-line gefitinib and BBR combination therapy in EGFR-mutant LUAD was evaluated; however, data regarding progression-free survival have not been disclosed (NCT03486496, clinicaltrials.gov). Isoflavone puerarin and puerarin 6”-O-xyloside, natural compounds extracted from *Pueraria Iobata*, have demonstrated substantial anticancer potency against numerous cancer cell types^[158-160]^. Findings suggested that initial doses of cisplatin nanoparticles showed efficacy by killing CAFs and inhibiting tumor growth; however, chronic exposure triggered paracrine Wnt family member 16 (Wnt16) secretion from CAFs, subsequently promoting tumor cell resistance in a stroma-rich bladder cancer model^[161]^. Nano-puerarin is reported to attenuate CAF activity through the suppression of reactive oxygen species (ROS) production in a murine model of triple-negative breast cancer^[162]^; and another study in LUAD, puerarin targets lung cancer stem-like cells through inhibition of AKT/c-Myc signaling^[163]^. Unfortunately, there are very few clinical trials about Puerarin to target CAFs and CSCs, and only one observational study investigates the potential therapeutic targets and underlying molecular mechanisms of puerarin in the context of giant cell tumor of bone giant cell tumors of bone (GCTB, NCT06331104, clinicaltrials.gov), and the results of this investigation have not been published.

Additional natural compounds, including ovatodiolide (OV), cinnamaldehyde (CA), curcumin (CUR), Resolvin D1 (RvD1), and minnelide, exhibit a significant impact on both CAFs and CSCs. OV, the bioactive component of Anisomeles indica, significantly reduces miR‐21‐5p levels within CSC-EVs, along with STAT3 and mTOR, suppresses CAF activation, and mitigates cisplatin resistance induced by CSC-EVs in OSCC^[89]^. In addition, OV treatment reduces exosomal cargoes, such as β-catenin, IL-6, TGF-β1, and signal transducer and activator of transcription 3 (p-STAT3), and overcomes 5-FU resistance in CRC^[9]^. CA, a central element of cinnamon, is reported to impede tumor growth by targeting Wnt/-catenin signaling^[164]^. Recent research in prostate cancer showed that CA induces mitochondrial-dependent apoptosis in CAFs via cytochrome c release, ROS overaccumulation, and caspase activation, reversible by glutathione supplementation^[165]^. Similarly, CUR, a bioactive polyphenolic compound isolated from *Curcuma longa rhizomes*, triggers ROS-mediated endoplasmic reticulum (ER) stress in prostate CAFs through the PKR-like ER kinase (PERK)-eukaryotic initiation factor 2 alpha (eIF2a)-activating transcription factor 4 (ATF4) axis, disrupting CAF-driven stromal remodeling^[166]^. In pancreatic cancer, superparamagnetic iron oxide nanoparticles-encapsulated CUR (SP-CUR) impedes the interaction between tumor and stromal cells by inhibiting the SHH pathway and the CXCR4/CXCL12 signaling. Orthotopic mouse models demonstrate that SP-CUR combined with GEM reduces CSC populations, attenuates stromal density, and modulates tissue biomechanics by decreasing ECM rigidity^[167]^. The potential of adjuvant curcumin to improve recurrence-free survival in prostate cancer patients following prostatectomy is under investigation in clinical trials (NCT02064673, clinicaltrials.gov). Moreover, an endogenous anti-inflammatory lipid mediator, RvD1, inhibits CAF-induced EMT and stemness features in cancer cells through the suppression of cartilage oligomeric matrix protein (COMP) secretion, mediated by formyl peptide receptor 2 (FPR2)-ROS-Forkhead box M1 (FOXM1) in HCC^[168]^. Minnelide, a plant-derived compound, demonstrated potent efficacy in deactivating CAFs and suppressing tumor growth via the TGF-β signaling pathway in preclinical models of pancreatic cancer^[169]^. By inhibiting MYC, minnelide attenuated tumor growth in preclinical medulloblastoma mouse models, leading to improved survival. Notably, it also potentiated the effects of adjuvant chemotherapy^[170]^. Minnelide significantly depletes the CD133^+^ CSC population in pancreatic cancer models^[171]^ and it selectively targets CSCs in human and murine TNBC cell lines, via inhibition of myelocytomatosis oncogene (Myc) and heat shock protein 70 (HSP70), with greater potency than in luminal subtype lines. Combining minnelide with chemotherapy led to a synergistic effect in vivo through immune reprogramming and enhanced cytotoxic T-cell infiltration^[172]^. The phase I study (NCT05566834) demonstrated clinically meaningful responses and a manageable safety profile for minnelide-paclitaxel salvage therapy in refractory advanced gastric cancer. Based on this positive signal, a phase II trial is now being launched to formally assess the effectiveness of the combination in the AGC setting^[173]^. These studies demonstrate the potential of natural products in targeting the crosstalk between CAFs and CSCs with low toxicity and high bioavailability. Although initial therapeutic outcomes are promising, clinical data on these therapies are still scarce.

Therefore, the therapeutic promise of natural compounds lies in their inherent polypharmacology - their ability to concertedly modulate multiple nodes within this resistant TME network, rather than inhibiting a single target. This systems-level intervention may be more effective in overcoming the redundancy and plasticity that render single-agent targeted therapies prone to failure. The current challenge is to move beyond phenomenological observations and precisely map the cause-effect relationships and potential synergies within this network to rationally design combination regimens for clinical translation.

Current clinical trials targeting the CAFs–CSCs crosstalk face several interconnected bottlenecks. A primary challenge is the lack of precise biomarkers for CAF subpopulations that maintain CSCs and drive therapy resistance. Commonly used markers such as FAP, α-SMA, and PDGFRα/β are not exclusive to CAFs, increasing the risk of off-target effects. Furthermore, the inherent complexity, heterogeneity, and dynamic plasticity of CAFs complicate the development of selective therapies. In early-phase trials, small-molecule inhibitors aimed at disrupting this crosstalk have often demonstrated insufficient efficacy or unforeseen safety issues, such as significant bleeding risk, failing to improve progression-free or OS in several studies.

To overcome these bottlenecks, potential solutions include a renewed focus on discovering and validating novel, context-specific CAF subpopulation markers to enable precise patient stratification and target engagement. Therapeutic strategies could shift toward multi-target approaches or combination therapies that concurrently address key pathways in both CAFs and CSCs, rather than single-agent inhibition. Additionally, repurposing or developing natural compounds (e.g., curcumin, puerarin) with multi-modal actions on the TME offers a promising, potentially safer avenue, though their clinical benefit requires validation in robust, well-designed trials. Ultimately, advancing this field will depend on integrating deeper biological insights with innovative trial designs that account for TME plasticity and heterogeneity.

## CONCLUSION AND CRITICAL VIEW

Therapeutically targeting isolated components of the TME often fails due to functional redundancy and adaptive resistance mechanisms^[174,175]^. CAFs exemplify this challenge: On the one hand, CAFs sustain CSC stemness through activation of pathways, such as STAT3, Wnt/-Catenin, Notch, and Hedgehog, while also reprogramming metabolism and remodeling the ECM to form a nutrient-rich and biomechanical niche that shelters CSCs from therapeutic insults. On the other hand, CSCs reciprocally activate CAFs via secreted proteins and exosome-communication, facilitating a self-reinforcing loop that enhances tumor plasticity and adaptability. Most existing CAF therapies focus on just one tumor-promoting factor, may only help in specific situations, and are unlikely to work for most patients; notably, they aim to inhibit or modify the CAFs themselves, not directly attack the tumor. This bidirectional crosstalk renders conventional therapies ineffective against stromal barriers and quiescent CSCs, driving therapeutic resistance. The dynamic crosstalk between CSCs and CAFs represents a critical axis in tumor progression and therapeutic resistance. This review addresses a critical and underexplored area in oncology: the interdependence of CSCs and CAFs in shaping tumor ecosystems. While prior studies have documented isolated roles of CAFs or CSCs, few have systematically explored their co-evolutionary signaling networks and niche dynamics. Unlike existing literature attempts to target CAF-specific biomarkers and the TGF- signaling or suppress stemness-associated pathways have faced limited clinical success. We emphasize that effectively disrupting the CSCs-CAFs crosstalk requires combinatorial strategies to overcome both CAF heterogeneity and CSC signaling redundancy.

Expediting drug development against CSCs-CAFs crosstalk can be achieved by repurposing existing anti-cancer drugs, either currently in clinical trials or FDA-approved, that simultaneously target both CSCs and CAFs. This repurposing strategy accelerates the development of therapies targeting CSCs-CAFs crosstalk, which capitalizes on the known safety profiles and tolerability of these existing drugs. Among the existing drugs, natural compounds demonstrate significant advantages in targeting the CSCs-CAFs crosstalk, as supported by the following evidence: The dual suppression of CSCs and CAFs directly inhibits CSC proliferation and viability, effectively blocking CAFs activation. They markedly decreased collagen deposition and other ECM components; regulated the key signaling pathways critical for CSCs maintenance and CAFs activity, such as the TGF-β signaling pathway, SHH pathway, the CXCR4/CXCL12 signaling, and STAT3.Targeting this axis holds therapeutic potential. Significant challenges remain, compounded by the scarcity of CSCs within the TME and the high heterogeneity of CAFs, which creates functional diversity in their crosstalk and leads to redundant signaling pathways. This is also the key factor why the molecular crosstalk or targeting mechanisms discussed in the review are not exclusive to the CSCs-CAFs axis.

Spatial transcriptomics, single-cell multiomics, and functional assays can map the heterogeneity of CSCs and CAF subtypes along with their spatiotemporal dynamics. Addressing the challenge of isolating rare CSCs, future breakthroughs will likely rely on multidisciplinary strategies that integrate engineering tools, such as microfluidics and materials science, with biological insights, including niche signaling and metabolic properties. It will also be essential to establish more robust functional validation systems, such as *in vivo* limiting dilution tumor formation assays, to ensure the acquired cells possess bona fide stem cell properties. The goal is to shift from labor-intensive isolation toward intelligent enrichment and even *in situ* analysis, thereby reducing reliance on conventional separation methods. At the same time, patient-derived organoids and 3D-bioprinted TMEs replicate stromal complexity. These insights are essential for developing precision therapies that target specific subgroups and molecules with pro-tumorigenesis or spatial functional specializations to disrupt the CSCs-CAFs crosstalk, thereby leveraging the CSC-CAF axis as a therapeutic vulnerability.

However, these models may not faithfully recapitulate the TME, owing to the absence of critical cell-cell interactions between cancer and stromal cells and the potential inadequacy of defined cytokine cocktails. Drug development targeting CSCs faces significant hurdles, including the technical difficulty of isolating these cells in vitro, the challenge of sustaining their stemness properties, and the need to account for the effects of TME. A high-content imaging screening of 3D coculture models to identify agents targeting CSCs and CAFs, thereby accelerating drug discovery. However, this TME-based drug screening platform is currently confined to low throughput, and only holds potential for stroma-rich cancers such as lung cancer, pancreatic cancer, and breast cancer. Besides, the poor solubility and low bioavailability of natural compounds may impede drug development in the clinic. To overcome challenges, further clinical studies are needed to investigate the use of nanocurcumin. Engineered nanotherapeutics show promise for dismantling CAFs’ barriers and selectively targeting CSCs with reduced toxicity. In addition, targeting CAFs-CSCs crosstalk can also convert tumor-supportive CAFs into inhibitory phenotypes through a “re-education” strategy, while using CAR-T cell therapy to target CSC-specific antigens. Overall, the CAFs-CSCs crosstalk represents a generalizable axis that opens new avenues for combination targeted therapy in solid tumors, potentially improving the management of chemoresistance and tumor progression. Additionally, adaptive clinical trials integrating real-time monitoring of stromal and CSC biomarkers could dynamically optimize treatment regimens, thereby bridging mechanistic insights to clinical translation.

## DECLARATIONS

### Copyright

© The Author(s) 2026.
